# Studies on California ants: a review of the genus *Temnothorax* (Hymenoptera, Formicidae)

**DOI:** 10.3897/zookeys.372.6039

**Published:** 2014-01-22

**Authors:** Roy R. Snelling, Marek L. Borowiec, Matthew M. Prebus

**Affiliations:** 1 Natural History Museum of Los Angeles County, 900 Exposition Boulevard, Los Angeles, California, 90007 U.S.A.; 2 University of California at Davis, 1 Shields Avenue, Davis, California, 95616 U.S.A.

**Keywords:** Taxonomy, identification, key, North America

## Abstract

The following ten new species of the ant genus *Temnothorax* are described and illustrated: *T. anaphalantus* (California, Baja California), *T. arboreus* (California), *T. caguatan* (Oregon, California, Baja California), *T. morongo* (California, Baja California), *T. myrmiciformis* (California, Baja California), *T. nuwuvi* (Nevada), *T. paiute* (California, Nevada), *T. pseudandrei* (Arizona, California), *T. quasimodo* (California) and *T. wardi* (California). A key to workers of the twenty-two *Temnothorax* species known or expected to occur in California is provided.

## Preface

An unfinished manuscript of this paper, by Roy R. Snelling, was handed down to the junior authors by Gordon Snelling, son of the late taxonomist. We feel grateful for this opportunity to see that one of Roy’s last contributions to hymenopteran systematics is published.

## Introduction

Below we describe ten new species of the ant genus *Temnothorax* Mayr, 1861. The material originates principally from California with additional specimens from Baja California, Nevada and Oregon. We also present a new key and taxonomic synopses to all *Temnothorax* species known to occur in California.

The ant genus *Temnothorax* was revived from synonymy with *Leptothorax* Mayr 1855 by Bolton (2003), who transferred nearly all of the North American species formerly placed in *Leptothorax* to *Temnothorax*.

Most of the North American species now placed in *Temnothorax* had been included in the subgenus *Myrafant*, M. R. Smith 1950 and a few were members of the subgenera *Dichothorax*, Emery 1895 and *Macromischa*, Roger 1863. Bolton (2003) treated all of these names as synonyms of *Temnothorax*.

Recent molecular phylogenetic studies show that the genera *Chalepoxenus*, *Myrmoxenus* and *Protomognathus* are nested within *Temnothorax*, and that the latter is distinct from the more distantly related genera *Formicoxenus*, *Leptothorax* and *Harpagoxenus* (Beibl et al. 2007, Prebus in prep., Ward et al., unpublished data). Species in these ‘satellite’ genera live as social parasites within the nests of other species of *Temnothorax* (Buschinger 2009).

The North American species of the erstwhile subgenus *Myrafant*, which includes all but a few of our California species, were revised by Mackay (2000), who recognized 11 species in California, five described as new. Three of those species have subsequently been placed in synonymy (Ward 2005). Ward (2005) listed 20 species of *Temnothorax* in California, ten of them undescribed at that time. Nine of those ten species are described below; based on examination of *Temnothorax neomexicanus* specimens from Arizona, Nevada, California and images of the syntype, we consider his tenth species, *Temnothorax* sp. CA-10, to represent a variant of *Temnothorax neomexicanus*, differing by slightly larger body size (*Temnothorax* sp. CA-10 workers, 5 measured, WL = 0.804–0.865 (0.826) vs. *Temnothorax neomexicanus* workers, 6 measured, WL = 0.634–0.814 (0.735); all measurements in mm). A few of the species described here also appeared under ‘species-codes’ in Johnson and Ward (2002).

At present, there are about 60 species (including those described below) of *Temnothorax* known from North America and more than 350 species worldwide (Fisher and Cover 2007, Bolton 2013), and so our study is of limited scope. Nevertheless, we believe that by officially describing these forms and giving a new illustrated key, we are providing a useful resource for myrmecologists working in western North America.

The workers of *Temnothorax* species are generally small. Colonies are typically monogynous, although facultative polygyny has been documented in several species (Alloway et al. 1983, Buschinger 1974, Buschinger and Linksvayer 2004, Rüppell et al. 1998). Colony populations are usually quite small, often with less than 100 workers (Beckers et al. 1989). However, several studies have found colonies of some species to be widely dispersed with several to many satellite nests (Alloway et al. 1983, Foitzik and Heinze 2001, Partridge et al. 1997). Many species are arboreal, living within hollow stems, old beetle or termite galleries, or in galls (Mackay 2000). *Temnothorax* species appear to be trophic generalists, feeding on a wide variety of scavenged items, including the elaiosomes of seeds (Espadaler 1997, Fokuhl et al. 2012). None have been documented to be active or aggressive predators.

## Methods

The specimens utilized in this study are principally from two collections, that of the University of California at Davis [UCDC] and the Natural History Museum of Los Angeles County [LACM]; additional samples were provided by B. E. Boudinot, Davis, California, R. A. Johnson, Tempe, Arizona, R. J. Hamton, Los Angeles, California, P. S. Ward, Davis, California [PSWC], and the authors M. L. Borowiec [MLBC] and M. M. Prebus [MMPC], both currently based in Davis, California. Type specimen images from the Museum of Comparative Zoology, Cambridge, Massachusetts [MCZC], Museo Civico di Storia Naturale, Genoa, Italy [MCSN] were also examined. Paratypes of most of the California species described by Mackay (2000) were donated to LACM by Dr. Mackay, University of Texas at El Paso [WPMC]. Types of the new species described here will be deposited in the aforementioned institutions, as well as the American Museum of Natural History, New York, New York, U.S.A. [AMNH], California Academy of Sciences, San Francisco, California, U.S.A. [CASC], and National Museum of Natural History, Washington, D.C., U.S.A. [USNM]. All specimen data for the new species, along with images, have been deposited on the AntWeb public database (http://www.antweb.org/) and are accessible through links to unique specimen identifiers in the text. The data can be also downloaded as an Appendix tab-delimited file.

The diagnoses accompanying the descriptions are intended to distinguish between the new species and others in the faunas of California and adjacent states, and not the North American fauna as a whole.

Where available, we provide illustrations and descriptions of general male morphology for the new species described here. However, the males of *Temnothorax* are still known for only a handful of taxa and we feel that detailed descriptions of genitalia and wing venation are not necessary for diagnostic purposes.

**Figure 1.**
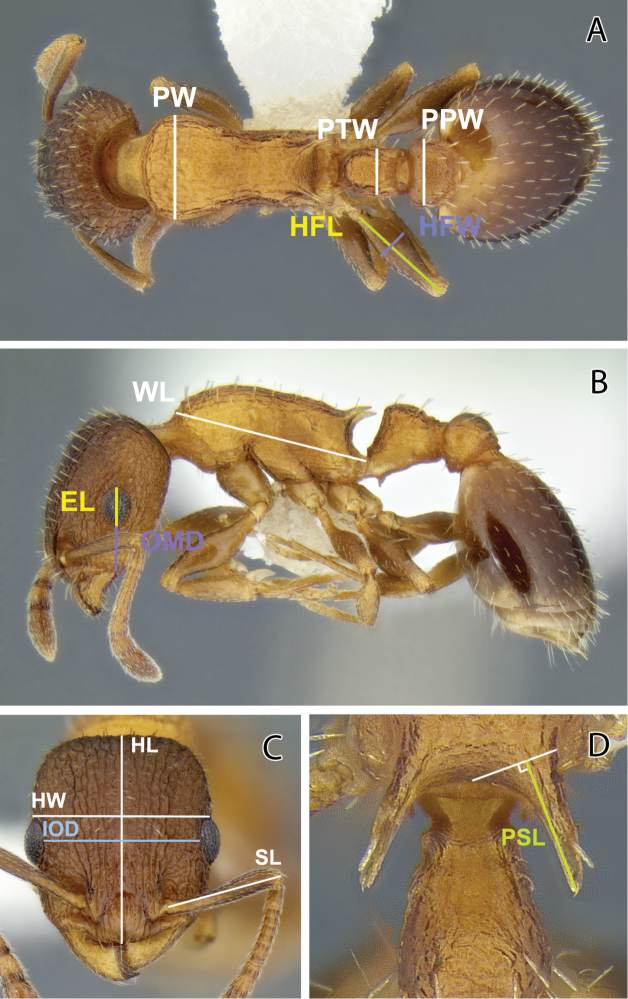
Images of *Temnothorax caguatan*, new species, illustrating the measurements used (CASENT0339286) **A** Body in dorsal view showing measuring lines for pronotum width (PW), petiole width (PTW), postpetiole width (PPW), hind femur length (HFL), and hind femur width (HFW) **B** Body in lateral view showing measuring lines for Weber’s, or mesosoma, length (WL), oculomandibular distance (OMD), and eye length (EL) **C** Head in full-face view showing measuring lines for head width (HW), head length (HL), scape length (SL), and head width (HW) **D** Dorsal view focusing on propodeal spines showing measuring line for propodeal spine length (PSL).

In the descriptions below, the following acronyms are used (Figure 1A–D):

EL Eye Length: Maximum length of the compound eye in profile, including non-pigmented facets.

HFL Hind Femoral Length: Maximum length of hind femur in dorsal view, excluding trochanter.

HFW Hind Femoral Width: Maximum width of hind femur in dorsal view.

HL Head Length: In frontal view, maximum length of head from anterior margin of clypeus to posterior margin of the head. Concave anterior clypeal and posterior head margins will reduce this measurement.

HW Head Width: Maximum width of head in frontal view, excluding eyes.

IOD Interocular Distance: Minimum distance between compound eyes in frontal view.

OMD Oculomandibular Distance (also called malar distance): Shortest length of malar area, i.e., the least distance between the anterior margin of the eye and the base of the mandible.

PPW Postpetiole Width: Maximum width of postpetiole in dorsal view.

PTW Petiole Width: Maximum width of petiole in dorsal view.

PSL Propodeal Spine Length: the tip of the measured spine, its base, and the center of the propodeal concavity between the spines must all be in focus. Using a dual-axis micrometer, the spine length is measured from the tip of the spine to a virtual point at its base where the spine axis meets orthogonally with a line leading to the median point of the concavity (Güsten et al. 2006).

PW Pronotal Width: Maximum width of pronotum in dorsal view.

SL Scape Length: Maximum straight-line length of scape shaft exclusive of basal condylar bulb and shaft.

WL Weber’s Length: Diagonal length of mesosoma in profile, from anterior declivity of pronotum (exclusive of pronotal ‘‘neck’’) to apex of metapleural lobe.

Indices:

CI Cephalic Index: (HW/HL) (100).

FI Femoral Length Index: (HFL/HW) (100).

OI Ocular Index: (EL/HL) (100).

PSI Propodeal Spine Index: (PSL/HL) (100).

PI Petiole index: (PPW/PTW) (100)

SI Scape Index: (SL/HL) (100).

All measurements are given in millimeters. Ranges are followed by mean in parantheses. All measurements and color photographs were made using a Leica MZ 16 stereomicroscope with a JVC digital video camera. Images were processed using Syncroscopy Automontage and Zerene Systems Zerene Stacker software and cleaned and adjusted using Adobe Photoshop. Maps were generated with ArcMap. In the material examined sections GPS coordinates have been rounded off to reflect the precision with which a record has been georeferenced or are followed by an error term in meters (e.g., 37.23100°, -122.10853° ±440 m).

## Results

### Temnothorax anaphalantus sp. n.

http://zoobank.org/77DCBF34-5B2B-476E-B45C-ED4C0210096C

http://species-id.net/wiki/Temnothorax_anaphalantus

**Figure 2.**
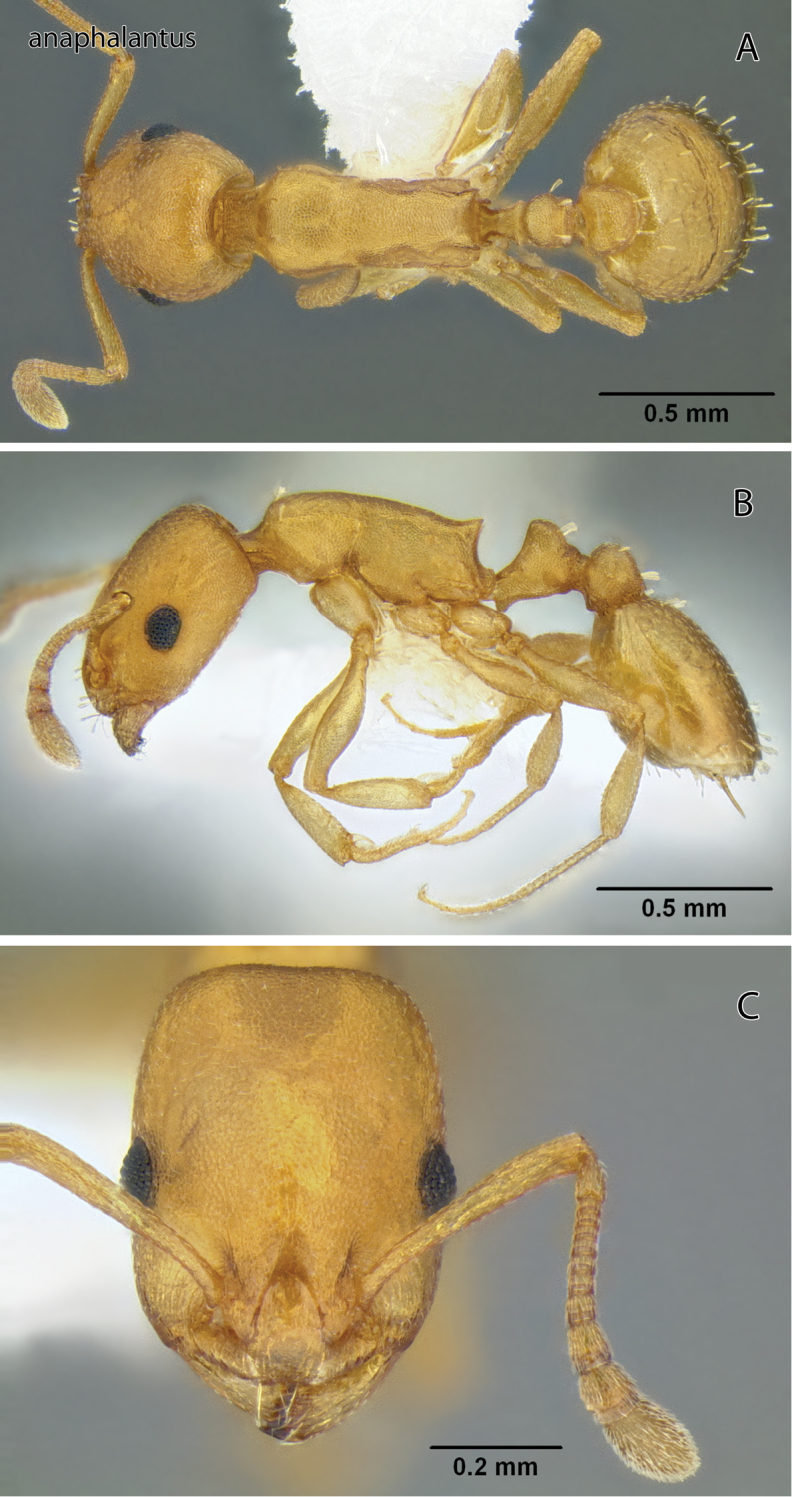
*Temnothorax anaphalantus* sp. n., worker (CASENT0339237) **A** Body in dorsal view **B** Body in lateral view **C** Head in full-face view.

**Figure 3.**
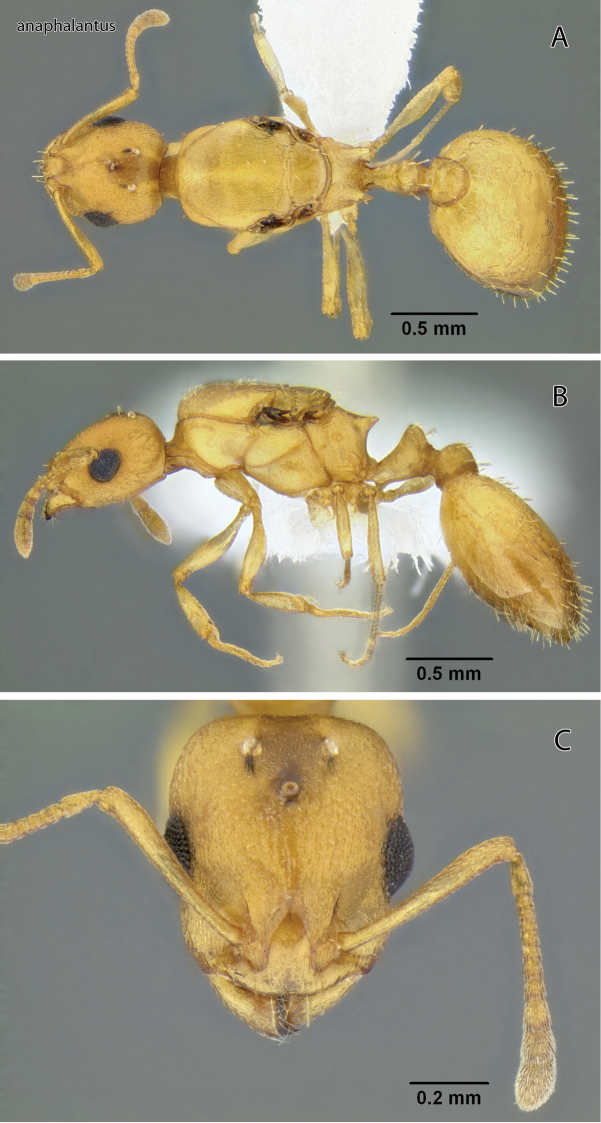
*Temnothorax anaphalantus* sp. n., dealate gyne (CASENT0339237) **A** Body in dorsal view **B** Body in lateral view **C** Head in full-face view.

**Figure 4.**
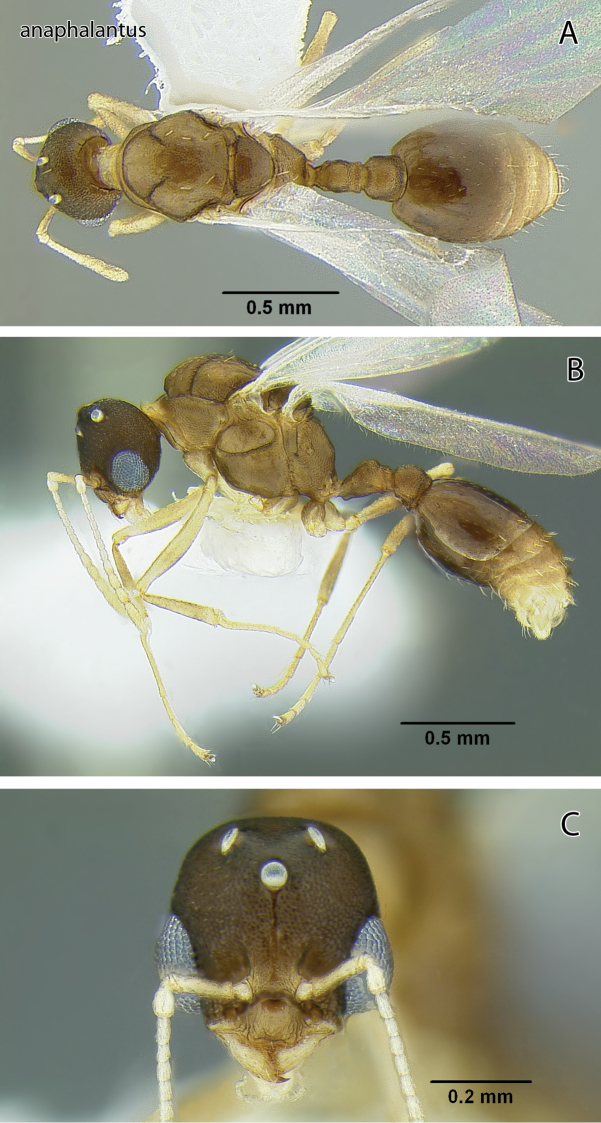
*Temnothorax anaphalantus* sp. n., male (CASENT0339242) **A** Body in dorsal view **B** Body in lateral view **C** Head in full-face view.

Figures 2 – 4 , 19 , 23 [Fig F24] [Fig F25] –26

Temnothorax sp. CA-06; Ward 2005: 68.

#### Diagnosis of worker.

Head and mesosoma dull and reticulate; erect setae absent from frons and vertex; mesosomal dorsum with about 12 short stout setae; propodeal spines thick at base; petiole node rounded in profile; body color yellowish to light brown.

#### Description.

Worker measurements (mm) (8 measured): EL 0.128–0.161 (0.145); HFL 0.433–0.486 (0.463); HFW 0.095–0.110 (0.104); HL 0.596–0.670 (0.647); HW 0.456–0.537 (0.505); IOD 0.395–0.457 (0.436); OMD 0.137–0.153 (0.147); PPW 0.200–0.229 (0.216); PSL 0.077–0.114 (0.100); PTW 0.147–0.170 (0.163); PW 0.299–0.350 (0.330); SL 0.419–0.463 (0.440); WL 0.662–0.746 (0.715). Indices: CI 76.5–80.3 (77.9); FI 88.3–95.0 (91.8); OI 21.4–24.3 (22.6); PI 125–138 (133); PSI 14.4–17.0 (15.5); SI 65.3–70.6 (67.9).

Head longer than broad in frontal view, lateral margins distinctly convergent above; posterior margin weakly concave in middle. Antenna 12-segmented; scape ending below posterior margin by more than twice its apical width; terminal club distinctly three-segmented. Eye small and moderately bulging; IOD 2.81–3.12 × EL; EL 0.93–1.10 × OMD. Median lobe of clypeus without submedian carinae. Head, including median lobe of clypeus and supraclypeal area, dull and finely reticulate; very fine rugae curving around antennal fossae and extending a short distance back from frontal lobes. Vertex, frons and frontal lobes without erect setae; clypeus with a single erect seta on each side of median lobe in addition to usual long curled setae along anterior margin.

Mesosoma slender, WL 2.13–2.21 × PW; in profile, pronotum sloping slightly down from mesonotum, mesonotum plus metanotum nearly flat. Propodeal spines elongate-triangular with acute tips, PSI 14.4–17.0, nearly as long as distance between their bases. Entire mesosoma dull and finely reticulate; several very short cross-rugae present on lower metapleural suture and several longer rugae anterior to metapleural gland. Metafemur 4.28–4.63 times longer than wide in dorsal view. Erect setae distinctly flattened, with 4 anteriorly across pronotum, 2 anteriorly and 2 posteriorly on mesonotum+metanotum, 1 on each propodeal spine.

Petiole node highest anteriorly, rounded dorsal face sloping distad into posterior face; subpetiolar process short and thick. Postpetiole rounded in profile and quadrate in dorsal view. Both nodes dull and finely reticulate, without rugae and each with several flattened erect setae that are longer than those of mesosoma.

Gaster, in dorsal view, 2.51–3.02 times wider than postpetiole; basal half of first tergite slightly shiny and distinctly roughened, becoming increasingly shiny and more weakly sculptured distad; all segments with scattered short, erect or suberect flattened setae.

Head and body uniformly pale brownish yellow to light brown, gaster darker posteriorly.

Gyne measurements (1 measured): EL 0.212; HFL 0.614; HFW 0.110; HL 0.717; HW 0.594; IOD 0.496; OMD 0.150; PPW 0.289; PSL 0.146; PTW 0.203; PW 0.661; SL 0.531; WL 1.18. Indices: CI 82.8; FI 103; OI 29.6; PI 142; PSI 20.4; SI 74.1.

Head longer than broad, margins approximately parallel in frontal view and broadly rounded into transverse posterior margin. Antennal scape extending back to level of lateral ocellus. Eye large and moderately convex, IOD 2.34 × EL in frontal view; EL 1.41 × OMD. Sculpture and pilosity about as in worker but longitudinal rugae more pronounced and a pair of hairs arising anterior to lateral ocelli.

Mesosoma slender, WL 1.79 × PW. Mesonotum flat in profile. Propodeal spines extremely thick at base, PSI 20.4, about half as long as infraspinal distance. Mesoscutum reticulate with faint longitudinal rugae in posterolateral area; mesoscutellum reticulate. Side of pronotum, mesepisternum and propodeum, including declivity, reticulate without rugae. Pilosity suberect to erect, short (less than 0.1 mm), flattened and club-shaped.

Remainder as described for worker; gaster 3.17 times as wide as postpetiole.

Male measurements (2 measured): EL 0.200–0.205 (0.203); HFL 0.590–0.622 (0.606); HFW 0.074–0.077 (0.076); HL 0.443–0.460 (0.452); HW 0.411–0.442 (0.427); IOD 0.282–0.300 (0.291); OMD 0.027–0.030 (0.028); PPW 0.189–0.190 (0.190); PSL N/A; PTW 0.135–0.139 (0.137); PW 0.425–0.466 (0.446); SL 0.170–0.177 (0.174); WL 0.891–0.927 (0.909). Indices: CI 92.8–96.1 (94.4); FI 141–144 (142); OI 43.5–46.3 (44.9); PI 1.37–1.40 (1.38); PSI N/A; SI 38.4–38.5 (38.4).

Head longer than broad, margins behind eyes slightly convergent and broadly rounded into weakly convex posterior margin. Eyes large and strongly bulging; IOD 1.38–1.50 × EL; OMD very short, about equal to transverse diameter of anterior ocellus; interocellar distance 2.30–2.70 and ocellocular distance 2.60–2.70 times diameter of anterior ocellus. Antennae 13-segmented. Scape more than half of IOD. Head uniformly punctate, with fine rugae present on clypeus and in the area between antennal fossae and compound eye, extending from lateral clypeal margin to approximately 1/2 length of compound eye. Pilosity same as in worker, but with clypeal setae barely surpassing the clypeal margin in full face view.

Mesosoma robust, WL 1.99–2.10 × PW. Propodeal spines absent. Sculpture punctate, weakening to fine reticulation on mesopleuron and dorsal surfaces of mesonotum. Mesoscutum with two pairs of erect short, blunt setae; several pairs of similar setae along the lateral and posterior margins of mesoscutellum.

Petiole subtriangular, with petiolar node rounded in profile; subpetiolar process a small rounded lobe, approximately half as high as length of propodeal lobes.

Gaster 2.82–3.09 × PPW in dorsal view. Uniformly smooth and shiny except for traces of fine reticulation on the anterior margin of first tergite. Sparse decumbent pilosity present on the first gastric sclerites, with several short, erect, blunt tipped setae along the posterior margins of all gastric sclerites.

Mesosoma and petiolar segments light brown; appendages light brownish yellow; head and gaster reddish brown.

#### Material examined.

Holotype worker, U.S.A.: CALIFORNIA: Los Angeles Co.: Baldy Road, 34.23°, -117.66°, 5.vii.1992 (G. C. Snelling) at public restrooms (LACMENT299332) [LACM].

Paratypes. Same data as holotype, 1 worker (LACMENT299331) [LACM], 1 worker (LACMENT299330) [MCZC].

Non-paratypic material examined. U.S.A.: CALIFORNIA: Orange Co.: Chino Hills, 114 m, 33.886°, -117.657°, 1–31.i.2001 (R. N. Fisher) 1 worker (CASENT0005695) [LACM]; Los Angeles Co.: San Gabriel River, 640 m, 34.2592°, -117.8562°, 27.x.2005 (J. des Lauriers) buckwheat/sage chaparral, 1 dealate gyne, 1 worker (CASENT0339237) [UCDC]; San Diego Co.: Santa Ysabel Ecological Reserve, 920 m, 33.1281°, -116.6711°, 8.i.2007 8/31/2007 (R. N. Fisher) 1 worker (CASENT0339238) [UCDC]; San Bernadino Co.: 28km NW San Bernadino, 1000 m, 34.32139°, -117.45914° ±30 m, 17.iii.2011 (B. E. Boudinot, #BEB364-4) nest in dead shrub stump, montane chaparral, 1 dealate gyne, 1 worker (CASENT0339239) [MMPC, 2 workers (CASENT0339240) [MMPC]; MEXICO: BAJA CALIFORNIA: Rio San Jose, 1.6 km N Meling Ranch, 640 m, 30.9833°, -115.7500°, 16.v.2000 (R. A. Johnson, #RAJ2004) cottonwood riparian, 2 workers, 1 male (CASENT0339241) [UCDC], 2 workers, 1 male (CASENT0339242) [UCDC].

#### Etymology.

From Greek: anaphalantos, with a bald forehead, in allusion to the lack of erect setae on the vertex and posterior areas.

#### Remarks.

The reticulate head and mesosoma, without any obvious rugae, and the greatly reduced standing pilosity will separate this from any other western species. In Mackay’s key (2000) it will run to *Temnothorax silvestrii* from Arizona, but differs from both *Temnothorax silvestrii* and *Temnothorax morongo* (described below) by the greatly reduced pilosity, lack of rugae, and the much thicker, short propodeal spines (Figures 19A, D).

Little is known of the ecology of this species. Some specimens were collected from a pitfall sample in an area that is mostly oak grassland but including some coastal scrub and riparian scrub. Other records are from chaparral and a cottonwood riparian area. The only nest sample of *Temnothorax anaphalantus* was collected from dead wood in montane chaparral.

### Temnothorax andrei

(Emery, 1895)

http://species-id.net/wiki/Temnothorax_andrei

Figure 21

Leptothorax (Leptothorax) andrei Emery 1895: 322, pl. 8, fig. 15. Holotype worker, Martinez, California (Turner) [MCSN] [Images of CASENT0904748 holotype examined].
Leptothorax nitens var. *Heathii*Wheeler 1903: 245. Syntype workers, Pacific Grove, California [MCZC] [Not examined]. Synonymy by Ward 2005: 15.
Leptothorax nitens subsp. *occidentalis*Wheeler 1903: 245. Syntype workers, Friday Harbor, Washington [MCZC] [Not examined]. Synonymy by Ward 2005: 15.
Leptothorax ocellatus Mackay 2000: 383. Holotype worker, 5 mi W Mineral, Tehama Co., California, 4250’ (D. Chandler) [MCZC] [Not examined]. Synonymy by Ward 2005: 15.
Temnothorax andrei (Emery); Bolton 2003: 271. First combination in *Temnothorax*; Ward 2005: 15. See also.
Temnothorax ocellatus (Mackay); Bolton 2003: 272. First combination in *Temnothorax*.

#### Material examined.

298 series from California, 5 series from Nevada, 7 series from Arizona, 1 series from Colorado [MLBC, MMPC, PSWC, UCDC].

#### Remarks.

See Ward (2005) for a discussion of this species.

### Temnothorax arboreus sp. n.

http://zoobank.org/3B57B3A0-7B21-4495-8AEA-B0CE583F16E6

http://species-id.net/wiki/Temnothorax_arboreus

**Figure 5.**
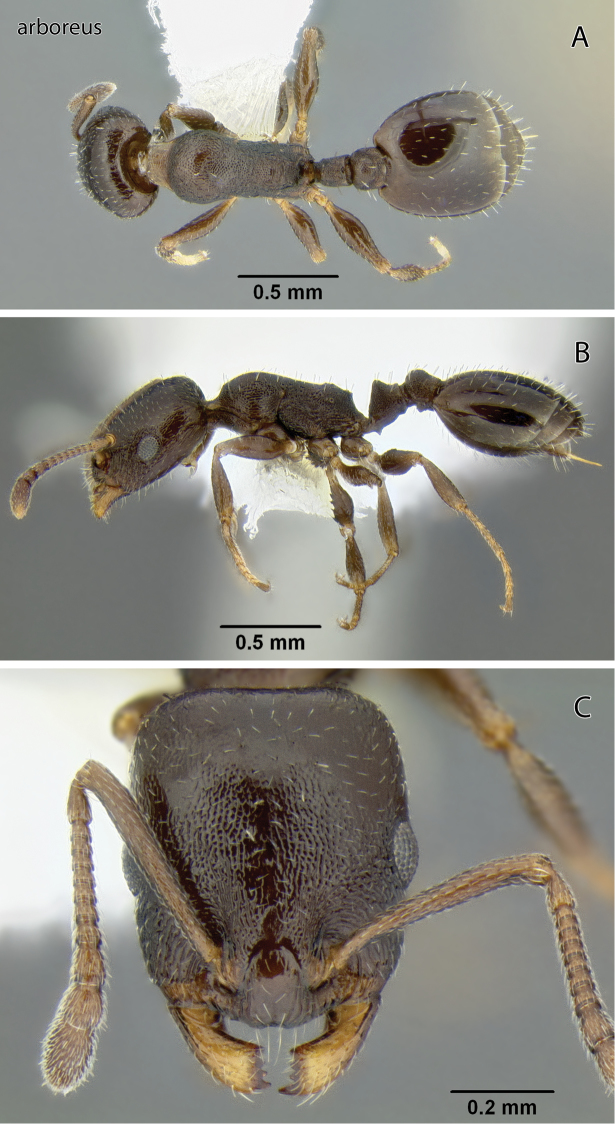
*Temnothorax arboreus* sp. n., worker (CASENT0339243) **A** Body in dorsal view **B** Body in lateral view **C** Head in full-face view.

Figures 5 , 19 , 24 – 26

Temnothorax sp. CA-09; Ward 2005: 68.

#### Diagnosis of worker.

Promesonotum flat, followed by sloping dorsal face of the propodeum, so that in lateral view propodeal angles are relatively low on the declivity; head shiny, varying from weakly sculptured to smooth; petiole node robust and triangular in profile; propodeal spines reduced to small angles; body color dark brown.

#### Description.

Worker measurements (mm) (4 measured): EL 0.128–0.142 (0.136); HFL 0.201–0.444 (0.371); HFW 0.101–0.123 (0.112); HL 0.584–0.652 (0.614); HW 0.475–0.534 (0.502); IOD 0.416–0.453 (0.435); OMD 0.151–0.187 (0.168); PPW 0.197–0.217 (0.206); PSL 0.025–0.034 (0.030); PTW 0.136–0.151 (0.143); PW 0.328–0.369 (0.347); SL 0.395–0.447 (0.425); WL 0.647–0.761 (0.702). Indices: CI 79.6–85.6 (81.8); FI 83.1–84.4 (83.9); OI 21.6–23.0 (22.1); PI 142–145 (144); PSI 4.28–5.21 (4.88); SI 67.6–71.3 (69.2).

Head longer than broad in frontal view, lateral margins very weakly curved, posterior margin flat to weakly curved. Antennal scape ending below posterior margin by more than its apical width; apical club distinctly 3-segmented. IOD 3.04–3.33 × EL; EL 0.75–0.91 × OMD. Mandible finely longitudinally rugose. Clypeus with 1 or 2 short fine carinae on either side of median carina. Dorsum of head shiny, mostly smooth between scattered fine piligerous punctures; area around frontal carinae moderately shiny and weakly reticulate; malar area with several short fine longitudinal rugae, interspaces dull and sharply reticulate; posterolateral angles of head moderately shiny and weakly reticulate. Dorsum of head with sparse short, fine setae.

Mesosoma slender, WL 1.97–2.11 times longer than PW; mesosomal dorsum nearly flat in profile before sloping down to obtuse propodeal denticles. Mesosomal dorsum moderately shiny and weakly reticulate but pronotum posteriorly with transverse smooth band; side of pronotum moderately shiny and irregularly rugulose, interspaces inconsistently reticulate; posterior face of propodeum dull and reticulate. Metafemur 3.54–3.97 times longer than wide in dorsal view. Entire length of dorsum with >20 flattened setae that are distinctly longer than those of frons.

Petiole without anterior peduncle; node robust and subtriangular, no higher than thick, summit subacute; subpetiolar tooth short and acute. Postpetiole profile low; in dorsal view node 1.42–1.45 times width of petiole node. Both segments moderately shiny and mostly finely reticulate. Each node with 4–6 setae similar to those of mesosoma.

Gaster in dorsal view 2.86–3.02 times wider than postpetiole; disc of first tergite smooth and shiny between sparse piligerous punctures; disc with sparse suberect flattened setae similar to those of mesosoma and scattered finer prostrate pubescence.

Head and body dark brown, gaster darker posteriorly.

Gyne and male unknown.

#### Material examined.

Holotype worker, U.S.A.: CALIFORNIA: San Diego Co.: Wooded Hill, 3 km SW Mount Laguna, 1800 m, 32.85°, -116.43°, 26.v.2004 (P. S. Ward #15254), on low vegetation [beating on *Pinus jeffreyi* foliage], *Pinus jeffreyi* forest (CASENT0339243) [UCDC].

Paratypes. Same data as holotype, 1 worker (CASENT0339243) [PSWC], 2 workers (CASENT0339244) [UCDC].

Non-paratypic material examined. U.S.A.: CALIFORNIA: Riverside Co.: San Jacinto Mountains, Idyllwild, 1610 m, 33.7°, -116.7°, 10.vii.1962 (C. O. Eads) on trunk of *Pinus jeffreyi*, 4 workers (CASENT0005933, LACMENT299357, LACMENT299356, LACMENT299355) [LACM].

#### Etymology.

From Latin, *arboreus* – of trees, in reference to this being an arboreal species.

#### Remarks.

This species is similar to *Temnothorax nitens* (Emery 1895) and in the key by Mackay (2000) it will run, with some difficulty, to *Temnothorax melinus* (Mackay 2000), a synonym of *Temnothorax nitens* (Ward 2005). From *Temnothorax nitens* it differs in the much darker color, the obtuse propodeal denticles that are shorter than the subpetiolar tooth, and the considerably more robust petiole node (Figure 19B & 21E).

As the name suggests, *Temnothorax arboreus* is an arboreal species. Workers have been collected from the trunk and beaten from foliage of *Pinus jeffreyi*. That this ant is arboreal further distinguishes it from *Temnothorax nitens*, a species that nests in soil, often under small covering objects such as stones.

A couple of collections of similar species are known from southeastern Arizona, but we feel the relationship of these samples will be better understood only when more material becomes available.

### Temnothorax caguatan sp. n.

http://zoobank.org/92EEDBB7-CF03-492D-A807-DAAC2662EA22

http://species-id.net/wiki/Temnothorax_caguatan

Figures 1 , 6 – 8 , 20 , 24 – 26

Leptothorax sp. BCA-3; Johnson and Ward 2002: 1023.
Temnothorax sp. CA-03; Ward 2005: 68.

#### Diagnosis of worker.

Antenna 11-segmented; propodeal spines distinct and more than half as long as distance between their bases; node of petiole node subrectangular in profile; head and mesosoma coarsely rugose and with reticulate interspaces.

#### Description.

Worker measurements (mm) (18 measured). EL 0.150–0.179 (0.162); HFL 0.461–0.582 (0.507); HFW 0.120–0.147 (0.129); HL 0.674–0.769 (0.706); HW 0.560–0.673 (0.604); IOD 0.486–0.585 (0.526); OMD 0.158–0.194 (0.171); PPW 0.257–0.321 (0.279); PSL 0.153–0.203 (0.166); PTW 0.192–0.259 (0.214); PW 0.387–0.480 (0.421); SL 0.437–0.524 (0.464); WL 0.757–0.927 (0.826). Indices: CI 81.8–88.5 (85.5); FI 79.7–90.5 (83.9); OI 21.7–24.4 (23.0); PI 123–138 (131); PSI 22.4–26.4 (23.5); SI 64.0–68.1 (65.7).

Head longer than broad in frontal view; lateral margins parallel or nearly so; posterior margin transverse. Antenna 11-segmented with indistinct apical 3-segmented club; scape ending below posterior margin by more than its apical width. Eye small; IOD 3.04–3.44 × EL; EL 0.88–1.04 × OMD. Mandibles coarsely longitudinally rugose. Median carina of clypeus flanked on each side by three about equally strong carinae. Dorsum of head with coarse longitudinal rugae that diverge slightly posteriorly; posterior one–third to one–half with coarse rugoreticulosity; interspaces slightly shiny with shallow punctures. Dorsum with sparse erect, stiff, blunt–tipped yellowish setae; ventral surface with several longer acute setae on each side.

Mesosoma slender, WL 1.91–2.03 × PW; profile of mesosomal dorsum nearly flat, sometimes weakly depressed at metanotal suture. Propodeal spines well developed, PSI 22.4–26.4, slightly down–curved in profile and about as long as distance between their bases. Dorsum and sides with strong, well–separated longitudinal rugae, interspaces contiguously punctate and slightly shiny; pronotum anteriorly usually with some reticulae. Metafemur 3.72–4.23 × longer than thick in dorsal view. Dorsum with 20+ short standing setae along entire length.

Petiole with anterior peduncle; node in profile subrectangular, dorsal face sloping posteriorly, flat to slightly convex; posterior face short; subpetiolar tooth prominent, usually acute; Postpetiole rectangular in dorsal view and 1.23–1.38 times as wide as petiole node. Petiole node rugoreticulate, postpetiole similar and with weak longitudinal rugae at sides; both nodes finely reticulate between rugae. Each with 6–10 standing setae that are longer than those of mesosoma.

Gaster, in dorsal view, 2.40–2.66 times wider than node of postpetiole. Disc of first tergum smooth and shiny between sparse, distinct piligerous punctures. All segments with numerous long, narrowly rectangular to subacute yellowish setae.

Head and body light to dark reddish-brown, mesosoma lighter than either head or gaster.

Gyne measurements (1 measured): EL 0.246; HFL 0.740; HFW 0.162; HL 0.811; HW 0.762; IOD 0.633; OMD 0.164; PPW 0.405; PSL 0.178; PTW 0.305; PW 0.814; SL 0.549; WL 1.446. Indices: CI 94.0; FI 97.1; OI 30.3; PI 133; PSI 22.0; SI 67.7.

Head longer than broad, margins approximately parallel in frontal view and broadly rounded into transverse posterior margin. Antennal scape extending back to level of lateral ocellus. Eye large and moderately convex, IOD 2.57 × EL in frontal view; EL 1.50 × OMD. Sculpture and pilosity about as in worker.

Mesosoma slender, WL 1.78 × PW. Mesonotum flat in profile. Propodeal spines thick at base, about half as long as infraspinal distance. Mesoscutum with conspicuous longitudinal rugae, and interspaces weakly reticulate; mesoscutellum also rugose, but more irregularly, underlying irregular sculpture more pronounced than on mesoscutum. Side of pronotum duller and with distinct fine reticulum; mesepisternum shinier and finely reticulate anteriorly to longitudinally rugose posteriorly; propodeum dull, finely reticulate with striae in anterior portion. Pilosity suberect to erect, short (less than 0.1 mm), not flattened and relatively sharp-tipped.

Remainder as described for worker; gaster 2.95 × as wide as postpetiole.

Male measurements (mm) (2 measured): EL 0.212–0.238 (0.225); HFL 0.686–0.695 (0.691); HFW 0.075–0.084 (0.080); HL 0.502–0.523 (0.513); HW 0.481–0.483 (0.482); IOD 0.333–0.376 (0.355); OMD 0.052–0.058 (0.055); PPW 0.221–0.225 (0.223); PSL N/A; PTW 0.173–0.181 (0.177); PW 0.641–0.668 (0.655); SL 0.140–0.150 (0.145); WL 1.044–1.150 (1.097). Indices: CI 92.4–95.8 (94.1); FI 143–144 (143); OI 40.5–47.4 (44); PI 124–128 (126); PSI N/A; SI 27.9–28.7 (28.3).

Head longer than broad, margins behind eyes slightly convergent and broadly rounded into weakly convex posterior margin. Eyes large and strongly bulging; IOD 1.57–1.58 × EL; OMD very short, about equal to transverse diameter of anterior ocellus; interocellar distance 2.30–2.40 and ocellocular distance 2.20–2.30 times diameter of anterior ocellus. Scape less than a third the length of IOD. Head finely reticulate and with regular longitudinal rugae dorsally and circling the eyes. Dorsum of head with erect setae which are long and sharp–tipped.

Mesosoma robust, WL 1.56–1.79 × PTW. Propodeal spines absent. Sculpture fine and weak, mesoscutal dorsum with very fine longitudinal rugosity, mesoscutellum mostly smooth with scattered rugae. Mesoscutum with scattered suberect short, slender setae; several pairs of longer setae submedially on mesoscutellum.

Summit of petiole node low, broadly convex in profile; subpetiolar process absent.

Mesosoma and petiolar segments brownish yellow; appendages yellow; head and gaster reddish brown.

**Figure 6.**
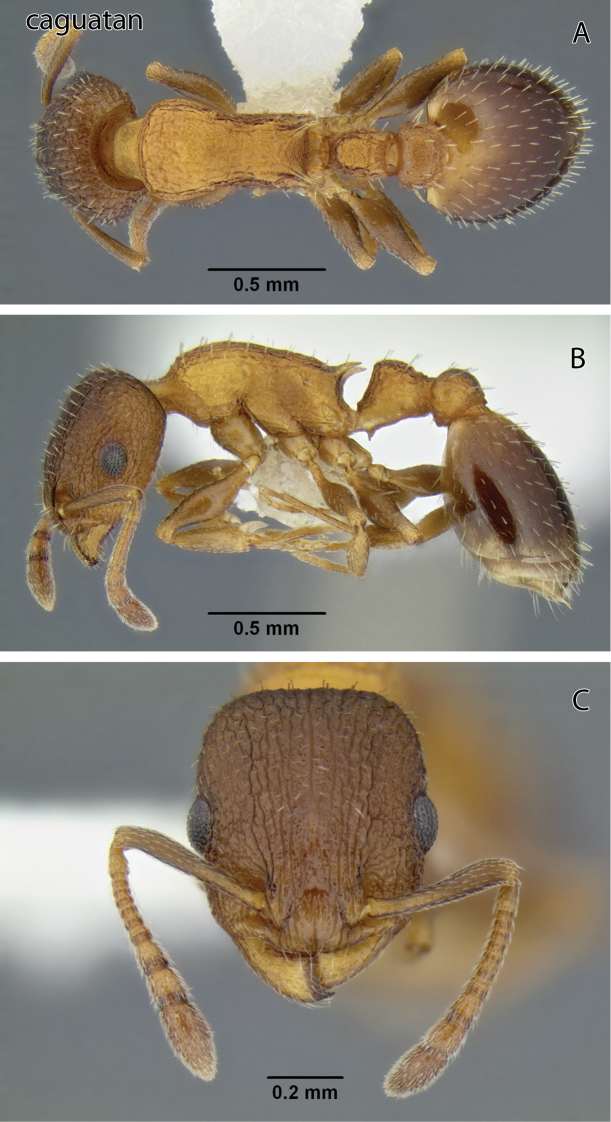
*Temnothorax caguatan* sp. n., worker (CASENT0339286) **A** Body in dorsal view **B** Body in lateral view **C** Head in full-face view.

**Figure 7.**
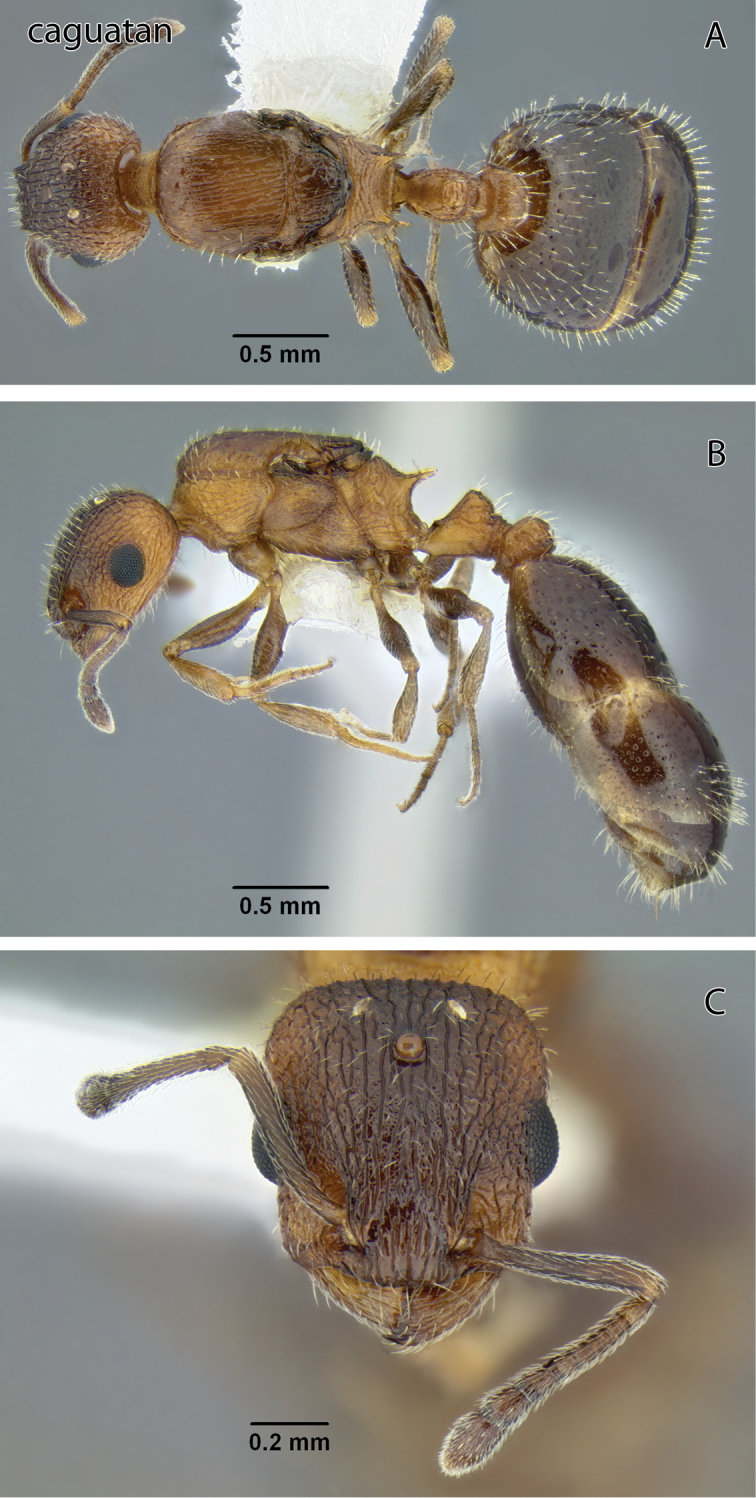
*Temnothorax caguatan* sp. n., dealate gyne (CASENT0339250) **A** Body in dorsal view **B** Body in lateral view **C** Head in full-face view.

**Figure 8.**
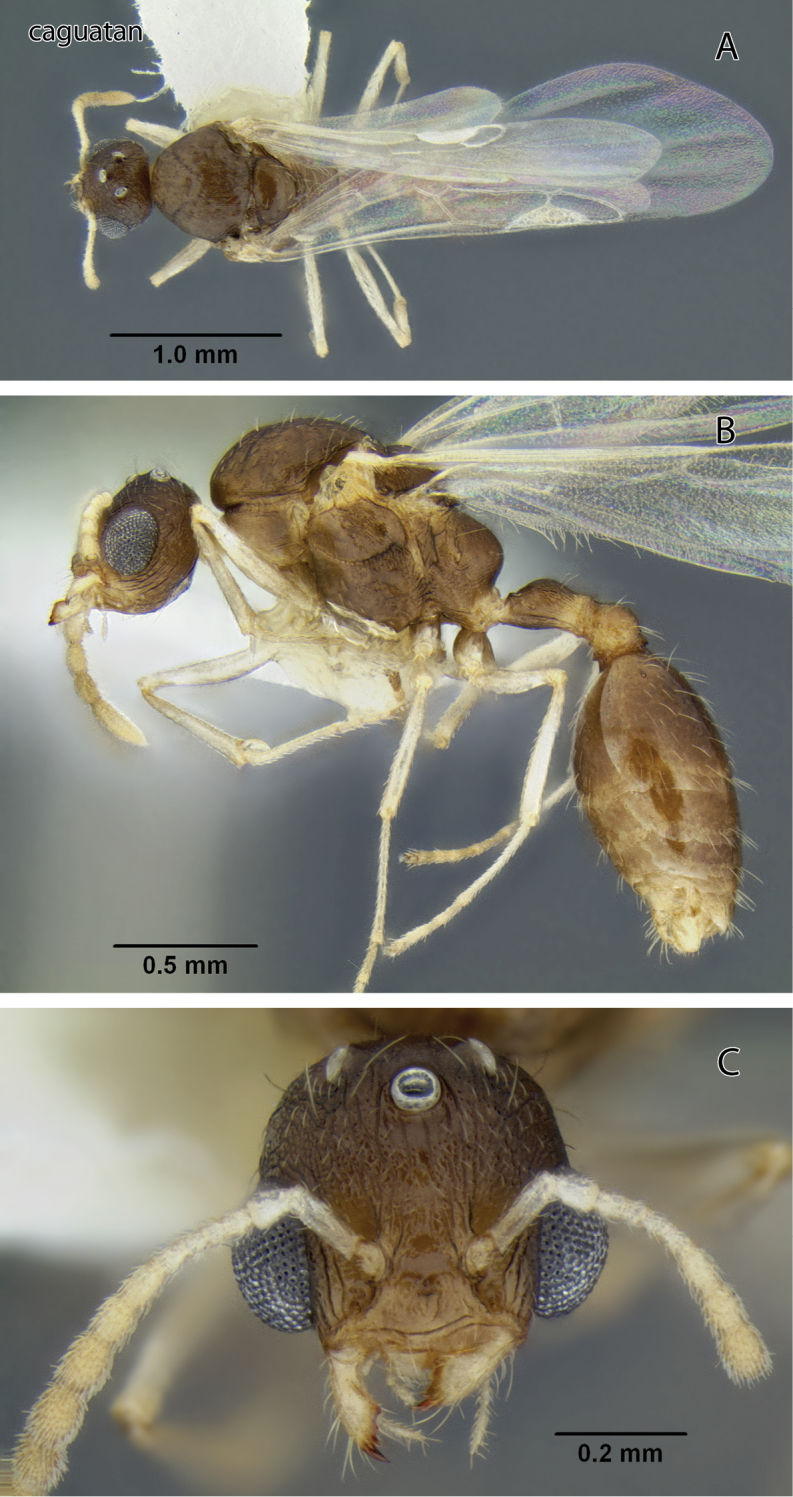
*Temnothorax caguatan* sp. n., male (CASENT0339304) **A** Body in dorsal view **B** Body in lateral view **C** Head in full-face view.

#### Material examined.

Holotype worker, U.S.A.: CALIFORNIA: San Diego Co.: Wooded Hill 3km SW Mount Laguna, 1800 m, 32.85°, -116.43°, 26.v.2004, (P. S. Ward, #15252), ex dead branch of *Pinus jeffreyi*, *Pinus jeffreyi* forest (CASENT0339251) [UCDC].

Paratypes. Same data as the holotype, 1 worker (CASENT0339252) [AMNH], 1 worker (CASENT0339253) [AMNH], 1 worker (CASENT0339254) [AMNH], 1 worker (CASENT0339255) [CASC], 1 worker (CASENT0339256) [CASC], 1 worker (CASENT0339257) [CASC], 1 worker (CASENT0339258) [LACM], 1 worker (CASENT0339259) [LACM], 1 worker (CASENT0339260) [LACM], 1 worker (CASENT0339261) [USNM], 1 worker (CASENT0339262) [USNM], 1 worker (CASENT0339263) [USNM]; 1 dealate gyne, 2 workers (CASENT0339250) [UCDC], 1 worker (CASENT0339264) [UCDC], 1 worker (CASENT0339265) [UCDC], 1 worker (CASENT0339266) [UCDC].

Non-paratypic material examined. U.S.A.: OREGON: Jackson Co.: Jacksonville, 560 m, 42.31°, -122.982°, 16.vi.2007 (P. S. Ward, #PSW15896.1) on *Arctostaphylos*, oak woodland, 3 workers (CASENT0106506) [UCDC]; Josephine Co.: 6 km NW Kerby, 460 m, 42.23°, -123.70°, 30.vii.1999 (P. S. Ward, #PSW14130-1) on low vegetation, *Pinus jeffreyi* forest, 1 worker (CASENT0339281) [UCDC]; Napa Co.: 5 km W Oakville, 560 m, 38.4300°, -122.4500°, 1.iv.1994 (P. S. Ward, #PSW12504-3) on *Arctostaphylos*, chaparral on serpentine, 3 workers (CASENT0100026) [UCDC]; Nevada Co.: Lang Crossing, South Yuba River, 1425 m, 39.32°, -120.65°, 21.iv.1994 (P. S. Ward, #PSW12570) ex dead branch of *Quercus chrysolepis*, oak woodland, 3 workers (CASENT0339245) [UCDC]; Nevada Co.: Lang Crossing, South Yuba River, 1425 m, 39.32°, -120.65°, 21.iv.1994 (P. S. Ward, #PSW12570) ex dead branch of *Quercus chrysolepis*, oak woodland, 3 workers (CASENT0339246) [PSWC]; Placer Co.: 2 km E Colfax, 490 m, 39.1°, -120.93°, 12.iv.1994 (P. S. Ward, #PSW12561) ground forager, oak woodland, 1 worker (CASENT0339247) [UCDC]; Placer Co.: 2 km E Colfax, 490 m, 39.1°, -120.93°, 12.iv.1994 (P. S. Ward, #PSW12562) on *Arctostaphylos*, oak woodland, 1 worker (CASENT0339248) [UCDC]; San Benito Co.: San Benito Mountain, 1320 m, 36.38°, -120.65°, 17.iv.1999 (P. S. Ward, #PSW13997-2) on *Arctostaphylos*, chaparral on serpentine, 2 workers (CASENT0339249) [UCDC]; San Mateo Co.: Jasper Ridge, 150 m, 37.4°, -122.23°, 27.iv.2003 (P. S. Ward, #PSW14825) ex dead branch of *Quercus agrifolia*, oak woodland, 3 workers (CASENT0339267) [UCDC]; San Mateo Co.: Jasper Ridge, 150 m, 37.40°, -122.23°, 27.iv.2003 (P. S. Ward, #PSW14832-3) on *Arctostaphylos*, chaparral, 2 workers (CASENT0100027) [UCDC]; Santa Barbara Co.: Los Padres National Forest, Cachuma Saddle, 1080 m, 34.73°, -119.92°, 27.xi.1986 (P. S. Ward, #PSW8780) ex dead branch of *Quercus agrifolia*, oak woodland, 1 dealate gyne, 18 workers (CASENT0339268, CASENT0339269, CASENT0339270, CASENT0339271, CASENT0339272, CASENT0339273, CASENT0339274) [UCDC]; Santa Barbara Co.: Los Padres National Forest, Ranger Peak, 1110 m, 34.72°, -119.97°, 27.xi.1986 (P. S. Ward, #PSW8786) on *Arctostaphylos*, oak woodland, 1 worker (CASENT0339275) [UCDC]; Santa Cruz Co.: Castle Rock State Park, 880 m, 37.2317°, -122.1083°, 18.iv.2010 (M. L. Borowiec, #MLB12) nest in dead branch on ground, oak woodland, 3 workers (CASENT0339308) [MLBC]; Tulare Co.: 15 km NE Three Rivers, 900 m, 36.52°, -118.77°, 17.iv.1994 (P. S. Ward, #PSW12557) on *Arctostaphylos*, oak woodland, 2 workers (CASENT0339276) [UCDC], 2 workers (CASENT0339277) [PSWC]; Plumas Co.: 8 km NW Quincy, 1030 m, 40.00°, -120.98°, 26.vi.1998 (P. S. Ward, #PSW13656) ex dead branch of *Quercus kelloggii*, mixed coniferous forest, 3 workers (CASENT0339278) [PSWC], 3 workers (CASENT0339279) [UCDC]; Plumas Co.: 8 km NW Quincy, 1030 m, 40°, -120.98°, 25.iv.2004 (P. S. Ward, #PSW15256) on tree trunk, mixed coniferous forest, 1 worker (CASENT0339280) [UCDC]; Santa Clara Co.: Blue Oak Ranch Reserve, 570 m, 37.382°, -121.738°, 22.xi.2009 (P. S. Ward, #PSW16285) ex dead branch of *Quercus agrifolia*, oak woodland, 3 workers (CASENT0106900) [UCDC]; Colusa Co.: 3 km W Fouts Springs, 1125 m, 39.35°, -122.7°, 22.iv.1985 (P. S. Ward, #PSW7483-4) on *Arctostaphylos*, chaparral, 2 workers (CASENT0339284) [UCDC]; Colusa Co.: 3 km W Fouts Springs, 1125 m, 39.35°, -122.7°, 22.iv.1985 (P. S. Ward, #PSW7483-4) on *Arctostaphylos*, chaparral, 2 workers (CASENT0339285) [UCDC]; CALIFORNIA: Colusa Co.: 1 km W Fouts Springs, 600 m, 39.3500°, -122.6667°, 14.v.1983 (P. S. Ward, #PSW5940-3) on *Arctostaphylos*, oak woodland, 2 workers (CASENT0339286) [UCDC]; Humboldt Co.: 3 km W Redway, 120 m, 40.1167°, -123.8500°, 25.vii.1981 (P. S. Ward, #PSW5096) on low vegetation, Sequoia forest, 1 worker (CASENT0339287) [UCDC]; Mariposa Co.: 7 km SW Yosemite Village, 1200 m, 37.7167°, -119.6500°, 19.vii.1981 (P. S. Ward, #PSW5081) ground forager, *Quercus*-*Pinus*-*Pseudotsuga* forest, 1 worker (CASENT0339288) [UCDC]; Mendocino Co.: 8km SW Hopland, 880 m, 38.93°, -123.20°, 2.vi.1984 (P. S. Ward, #PSW6767-1) on *Arctostaphylos*, *Cupressus* grove on serpentine, 3 workers (CASENT0339289) [UCDC]; Mendocino Co.: 8km SW Hopland, 880 m, 38.93°, -123.20°, 2.vi.1984 (P. S. Ward, #PSW6768-2) on low vegetation, *Cupressus* grove on serpentine, 3 workers (CASENT0339290) [UCDC]; Mendocino Co.: Hopland Field Station, 240 m, 39.0000°, -123.0833°, 25.vii.1981 (P. S. Ward, #PSW5089-1) on low vegetation, oak woodland, 1 worker (CASENT0005692) [UCDC]; Napa Co.: 9 km SE Knoxville, 240 m, 38.7500°, -122.2833°, 28.iv.1984 (P. S. Ward, #PSW6691) under stone, riparian woodland, 1 worker (CASENT0339291) [UCDC]; Nevada Co.: Lang Crossing, South Yuba River, 1425 m, 39.3167°, -120.6500°, 12.v.1984 (P. S. Ward, #PSW6732) on low vegetation, oak woodland, 2 workers (CASENT0339292) [UCDC], 2 workers (CASENT0339293) [PSWC]; Placer Co.: 25 km E Foresthill, 1370 m, 39.07°, -120.55°, 22.iv.1985 (P. S. Ward, #PSW7543) ex dead branch of *Quercus kelloggii*, *Quercus*-*Pinus*-*Pseudotsuga* forest, 3 workers (CASENT0339294) [UCDC]; San Diego Co.: Hwy. 94, 14.8 km E Campo, 965 m, 32.65°, -116.38°, 23.i.1995 (R. A. Johnson, #RAJCA95-1) in 1in dead branch of *Quercus agrifolia*, 3 workers (CASENT0339299) [UCDC], 3 workers (CASENT0339300) [PSWC]; San Diego Co.: 8 km N Descanso, 915 m, 32.9°, -116.64°, 1.iv.1972 30.iv.1972 (J. H. Hunt, #JHH621) in can trap, oak grove, 2 workers (LACMENT299346) [LACM]; San Diego Co.: 3 km S Santa Ysabel, 33.08°, -116.67°, 10.v.1987 (M. Trepanier) in dead branch of *Quercus virginiana* (sic), oak woodland, 3 workers (CASENT0339310) [UCDC]; San Diego Co.: 3 km S Santa Ysabel, 33.08°, -116.67°, 10.v.1987 (M. Trepanier) ground forager, chaparral/*Quercus virginiana* (sic) woodland, 1 worker (CASENT0339312) [UCDC]; San Diego Co.: 6 km W Julian, 33.08°, -116.66°, 10.v.1987 (M. Trepanier) on oak trunk, 2 workers (CASENT0339313) [UCDC]; San Diego Co.: 8 km N Descanso, 915 m, 32.9°, -116.64°, 12.iv.1972 (J. H. Hunt, #JHH869) 2 workers (LACMENT299352) [LACM]; Contra Costa Co.: Mt. Diablo summit, 1130 m, 37.8817°, -121.9167°, 22.iii.2008 (P. S. Ward, #PSW16052.1) sifted litter (leaf mold, rotten wood), oak woodland, 1 worker (CASENT0106644) [UCDC]; Nevada Co.: Empire Mine State Historic Park, near Grass Valley, 760 m, 39.21°, -121.04°, 24.iv.2011 (M. L. Borowiec, #MLB256.3) sifter leaf litter, mixed coniferous forest, 1 worker (CASENT0339307) [MLBC]; Santa Cruz Co.: Castle Rock State Park, 880 m, 37.23100°, -122.10853° ±440 m, 18.iv.2010 (P. S. Ward, #PSW16313) on tree trunk, oak woodland, 1 worker (CASENT0339301) [UCDC]; Riverside Co.: Kenworthy, 1425 m, 33.64339°, -116.62041° ±56 m, 28.iv.2011 (B. E. Boudinot, #BEB475) beating shrubs, chaparral on steep hill, 2 workers (CASENT0248048) [MMPC], 2 workers (CASENT0248049) [MMPC]; Mendocino Co.: 6.8 km SE Leggett, 455 m, 39.8337°, -123.6451°, 29.iii.2013 (M. M. Prebus, #MMP1066) on *Arctostaphylos*, arid slope, 1 worker (CASENT0339302) [MMPC]; Santa Barbara Co.: 7 km N Goleta, 610 m, 34.5°, -119.81°, 7.x.1984 (P. S. Ward, #PSW7095) ex dead branch of *Quercus agrifolia*, oak woodland, 1 male (CASENT0339303) [UCDC]; Santa Barbara Co.: 7 km N Goleta, 610 m, 34.51°, -119.81°, 7.x.1984 (P. S. Ward, #PSW7095) ex dead branch of *Quercus agrifolia*, oak woodland, 2 worker, 1 male (CASENT0339304) [UCDC]; MEXICO: BAJA CALIFORNIA: 20 km E Ensenada, 650 m, 31.88°, -116.40°, 27.iv.1998 (P. S. Ward, #PSW13581-1) on tree trunk, oak woodland, 3 workers (CASENT0339282) [UCDC], 3 workers (CASENT0339283) [UCDC]; Sierra San Pedro Martir, 24.3 km SW Observatorio, 1660 m, 30.97°, -115.60°, 8.ii.1996 (R. A. Johnson, #RAJBC96-22) in 0.5in dead branch of *Quercus agrifolia*, 3 workers (CASENT0339295) [UCDC], 3 workers (CASENT0339296) [UCDC]; Sierra San Pedro Martir, 24.3 km SW Observatorio, 1660 m, 30.97°, -115.60°, 8.ii.1996 (R. A. Johnson, #RAJBC96-23) in 0.5in dead branch of *Quercus agrifolia*, 3 workers (CASENT0339297) [UCDC]; Sierra Juarez, 15.3 km S Laguna Hanson, 1585 m, 31.93°, -115.97°, 15.v.2000 (R. A. Johnson, #RAJ2001) in 0.3in dead branch of *Quercus* sp., 3 workers (CASENT0339298) [UCDC].

#### Etymology.

When Hernán Cortéz was conquering central Mexico, the Nahua speaking people related to him tales of a fabulous land, ruled by women, far to the northwest that was rich in gold and gems. They named this land “Caguatán”, the Land of Women. This tale presumably inspired Cortéz and other avaricious conquistadors to search for this marvelous land, ultimately leading the Spaniards to the Californias. The name California is that used by some fiction writers and explorers of the time for a mythical land inhabited by beautiful black women ruled by their queen, Calafia.

#### Remarks.

Because the antennae are 11-segmented and the propodeal spines are long, among our California species *Temnothorax caguatan* can only be confused with *Temnothorax rugatulus*, which has a shorter petiole node with acute top and relatively more slender hind femur (Figure 20E, F; see key for measurements). The latter also typically nests in soil, commonly under covering objects such as small stones, although collections are known from dead wood and arboreal sites (P. S. Ward pers. comm.).

This widely distributed arboreal species has been collected in a variety of habitats, ranging from oak woodland to grey pine and Sequoia forests, but the preponderance of the records have been from oak woodland. It has been found in association with a number of tree and shrub species: *Arctostaphylos* spp., *Pinus jeffreyi*, *Quercus agrifolia*, *Quercus chrysolepis* and *Quercus kelloggii*. Nest samples have been taken from dead branches of all three *Quercus* species and one was from a dead branch of *Pinus jeffreyi*.

### Temnothorax carinatus

(Cole, 1957)

http://species-id.net/wiki/Temnothorax_carinatus

Figure 22

Leptothorax (Leptothorax) carinatus Cole 1957: 213, fig. 1. Holotype worker, 106 paratype workers, one paratype gyne, Limpia Canyon, Davis Mts., Texas [USNM] [Examined images of CASENT0105870 paratype worker].
Leptothorax carinatus Cole; Wheeler and Wheeler 1973: 70. Larva described.
Leptothorax (Myrafant) carinatus Cole; D. R. Smith 1979: 1392. First combination in *Leptothorax (Myrafant)*; Mackay 2000: 328. See also.
Temnothorax carinatus Cole; Bolton 2003: 271. First combination in *Temnothorax*.

#### Material examined.

2 series from Texas, 5 series from Mexico [PSWC, UCDC].

#### Remarks.

See Mackay (2000) for a full description of this species.

### Temnothorax chandleri

(Mackay, 2000)

http://species-id.net/wiki/Temnothorax_chandleri

Figures 22 –23

Leptothorax (Myrafant) chandleri Mackay 2000: 330, figs. 35, 99. Paratype worker, Red Bluff, Samson Slough, Tehama Co., California [WPMC] [Not examined]. Note: holotype and other paratype apparently lost; see Ward 2005: 19.
Temnothorax chandleri (Mackay); Bolton 2003: 271. First combination in *Temnothorax*; Ward 2005: 19. See also.

#### Material examined.

1 series from California [UCDC].

#### Remarks.

See Ward (2005) for a discussion of this species.

### Temnothorax gallae

(M. R. Smith, 1949)

http://species-id.net/wiki/Temnothorax_gallae

Figures 21 –22

Leptothorax (Leptothorax) gallae Smith 1949: 112. Holotype worker, Devil’s Gate Dam, Pasadena, California [MCZC] [Images of CASENT0105617 holotype worker examined].
Leptothorax (Myrafant) gallae (Smith); D. R. Smith 1979: 1393. First combination in *Leptothorax (Myrafant)*; Mackay 2000: 346. See also.
Temnothorax gallae (Smith); Bolton 2003: 271. First combination in *Temnothorax*.

#### Material examined.

45 series from California [MLBC, MMPC, PSWC, UCDC].

#### Remarks.

See Mackay (2000) for a full description of this species.

### Temnothorax morongo sp. n.

http://zoobank.org/3021AE9F-1152-4C4F-A24C-8FBE2B82F281

http://species-id.net/wiki/Temnothorax_morongo

**Figure 9.**
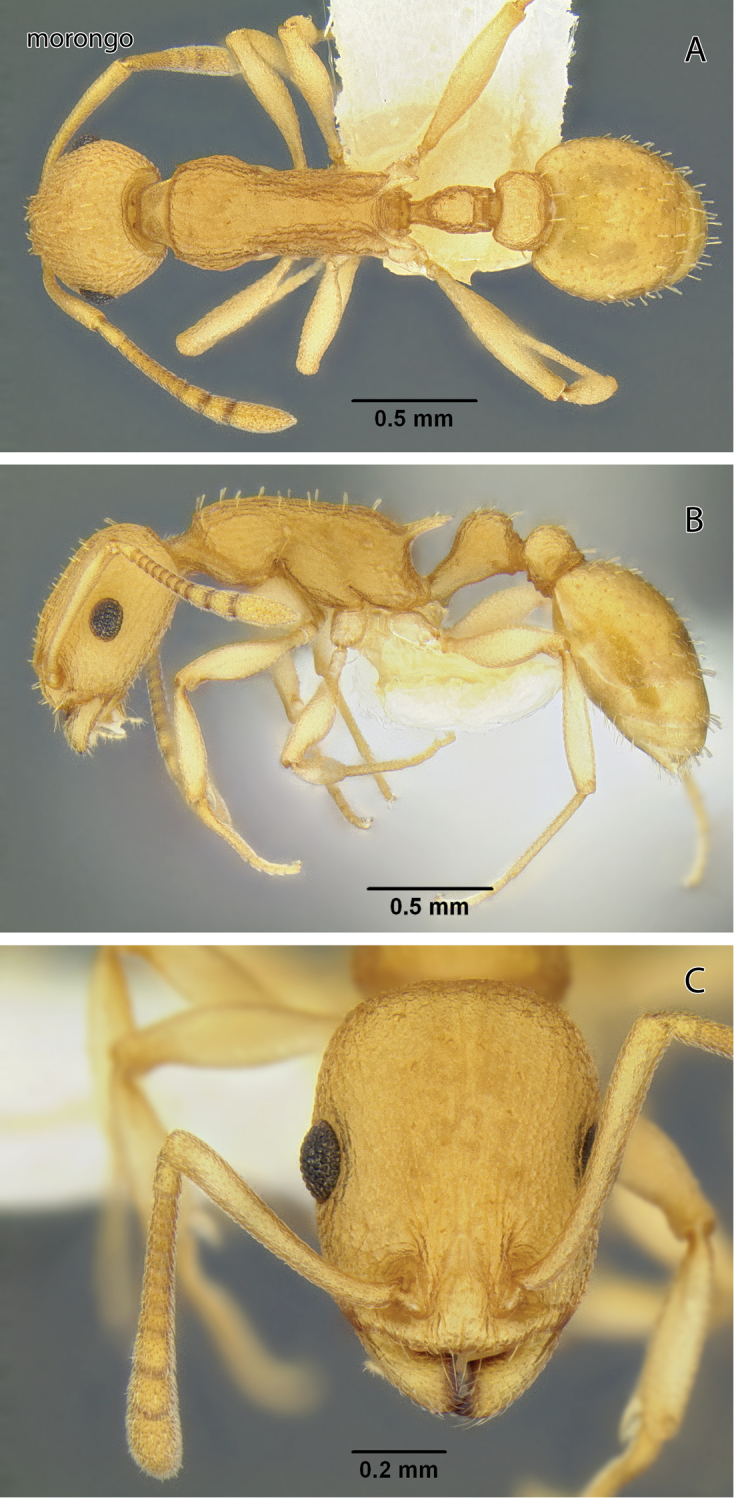
*Temnothorax morongo* sp. n., worker (CASENT0103108) **A** Body in dorsal view **B** Body in lateral view **C** Head in full-face view.

Figures 9 , 19 , 24 – 26

Leptothorax sp. BCA-1; Johnson and Ward 2002: 1023.
Temnothorax sp. CA-1; Ward 2005: 68.

#### Diagnosis of worker.

First gastral tergum of worker slightly shiny and finely reticulate, with sparse blunt stiff erect setae; mesosoma dull and contiguously punctate between widely spaced longitudinal rugae and pronotal dorsum with transverse anterior carina; propodeal spines long; postpetiole in dorsal view wide.

#### Description.

Worker measurements (mm) (6 measured): EL 0.152–0.218 (0.173); HFL 0.545–0.763 (0.652); HFW 0.116–0.151 (0.139); HL 0.659–0.87 (0.761); HW 0.537–0.718 (0.607); IOD 0.447–0.611 (0.506); OMD 0.182–0.237 (0.209); PPW 0.270–0.348 (0.302); PSL 0.155–0.227 (0.194); PTW 0.171–0.213 (0.191); PW 0.381–0.510 (0.433); SL 0.544–0.749 (0.627); WL 0.816–1.050 (0.931). Indices: CI 77.3–82.5 (79.8); FI 102–112 (107); OI 21.1–25.1 (22.7); PI 150–165 (158); PSI 22.2–28.3 (25.4); SI 79.5–86.1 (82.4).

Head longer than broad in frontal view; posterior margin transverse and lateral margins essentially parallel. Antenna 12-segmented; scape slightly exceeding posterior margin; apical club distinctly 3-segmented. Eye small, IOD 2.80–3.13 × EL; EL about 0.75–0.92 × OMD. Mandibles coarsely longitudinally rugose. Head opaque and finely reticulate between fine longitudinal rugae; interrugal spaces of clypeus shiny and weakly sculptured. Sparse yellowish very short and stout erect setae on front of head; hypostomal area with scattered very short fine setae.

Mesosoma slender, WL 2.06–2.24 × PW; mesosomal dorsum essentially flat in profile. Propodeal spines well developed, distinctly longer than distance between their bases; in profile directed distad or slightly down–curved; in dorsal view slightly incurved. Entire mesosoma opaque or nearly so between slightly irregular longitudinal rugae; anterior margin of pronotal disc with sharp transverse carina. Dorsum with sparse yellowish short, flattened setae. Metafemur slender, 3.98–5.32 times longer than wide in dorsal view.

Petiole node robust and cuboid in profile; acute subpetiolar tooth present. Postpetiole node rounded in profile; in dorsal view much broader than petiole node. Sculpture and pilosity of both nodes similar to those of mesosomal dorsum.

Gaster in dorsal view 2.01–2.29 times wider than postpetiole; slightly shiny and first tergum wholly finely reticulate and with sparse yellowish, slender suberect to erect setae.

Color of body yellowish to light orange, gaster and appendages slightly lighter.

Gyne and male unknown.

#### Material examined.

Holotype worker, MEXICO: BAJA CALIFORNIA: 19 km WNW Bahia de los Angeles, 28.98°, -113.75°, 1.vi.1997 (A. Suarez & T. J. Case), Sonoran desert, in pitfall trap (CASENT0339305) [UCDC].

Paratypes. Same data as holotype, 1 worker (CASENT0339305) [UCDC].

Non-paratypic material examined. U.S.A.: CALIFORNIA: Riverside Co.: Joshua Tree National Park, 33.8°, -116.0°, 28.v.1979 (E. L. Sleeper, #ELS2100) quail guzzler, 1 worker (CASENT0103108) [LACM]; Riverside Co.: Joshua Tree National Park, 33.8°, -116.0°, 25.vi.1966 (E. L. Sleeper) quail guzzler, 1 worker (LACMENT299328) [LACM]; Riverside Co.: Joshua Tree National Park, 33.8°, -116.0°, 9.vii.1966 (E. L. Sleeper) quail guzzler, 1 worker (LACMENT299329) [LACM]; MEXICO: BAJA CALIFORNIA SUR: Isla Carmen S end, 20 m, 25.86°, -111.22°, 13–18.vii.1999 (R. Aalbu, #ABRA11) 1 worker (CASENT0339306) [UCDC].

#### Etymology.

Named for the Morongo, a band of the Cahuilla people who occupied occupied the northern part of this species range, in what is now Joshua Tree National Park. The name is a noun in apposition.

#### Remarks.

In the keys of Creighton (1950) and Mackay (2000) *Temnothorax morongo* will run to *Temnothorax silvestrii* (Santschi 1911), an arboreal species from Arizona, which it resembles. In *Temnothorax silvestrii* the sculpture of the head and mesosoma is coarser, the head is proportionately a little broader, the propodeal spines are much shorter and the metafemora are much more robust, about three times longer than thick in dorsal view (also see Creighton 1953).

All known collections of this species are from pitfall traps, suggesting that this is a ground-nesting ant.

### Temnothorax myrmiciformis sp. n.

http://zoobank.org/BCBFE027-0E6D-48B1-AC1C-F28B0AE1FD43

http://species-id.net/wiki/Temnothorax_myrmiciformis

**Figure 10.**
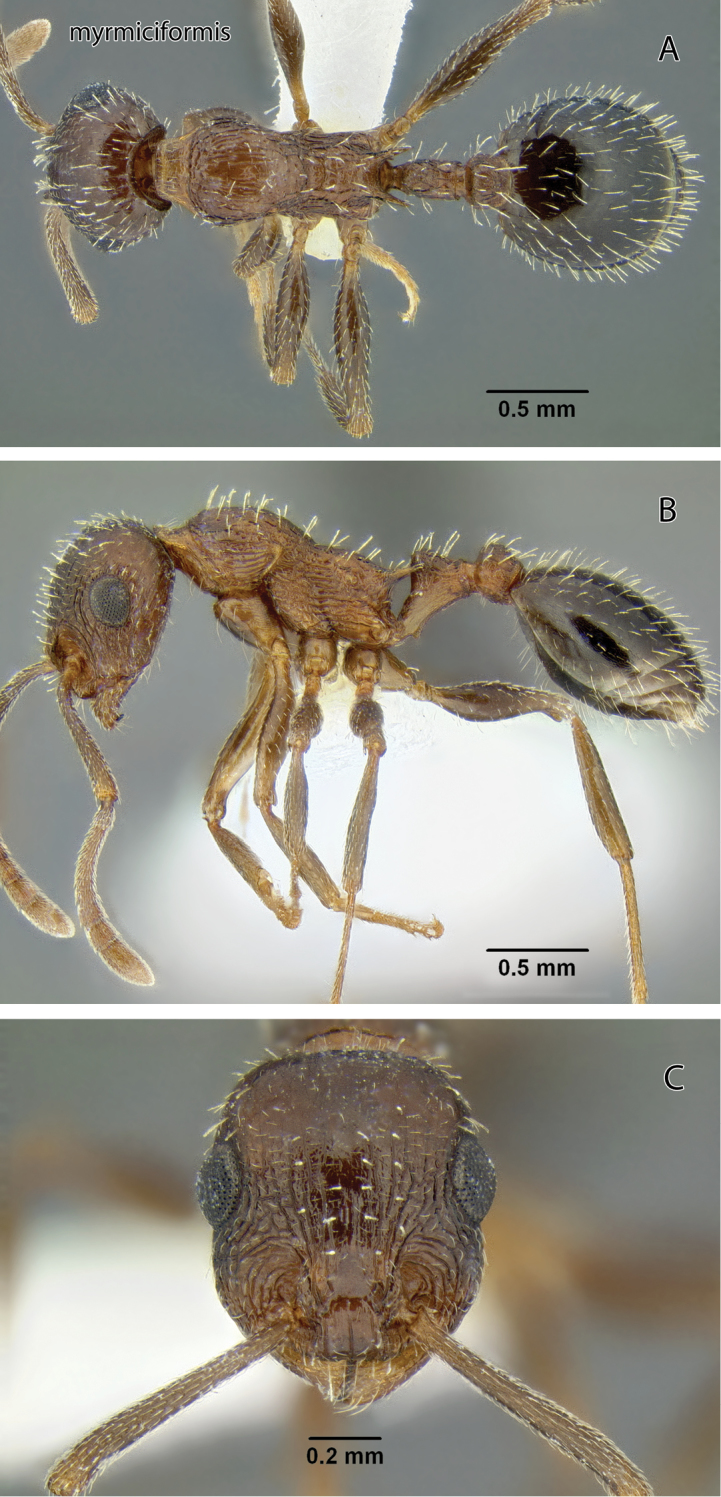
*Temnothorax myrmiciformis* sp. n., worker (CASENT0339323) **A** Body in dorsal view **B** Body in lateral view **C** Head in full-face view.

Figures 10 , 19 , 24 – 26

Leptothorax sp. BCA-9; Johnson and Ward 2002: 1024.
Temnothorax sp. CA-07; Ward 2005: 68.

#### Diagnosis of worker.

Large, gracile species with large eyes; scape extending well beyond posterior margin; dorsum of mesosoma evenly sloping from pronotum to base of propodeal spines; side of head behind eyes with numerous standing setae along margin.

#### Description.

Worker measurements (mm) (9 measured): EL 0.216–0.250 (0.217); HFL 0.759–0.908 (0.789); HFW 0.150–0.187 (0.164); HL 0.774–0.904 (0.821); HW 0.624–0.748 (0.665); IOD 0.525–0.636 (0.556); OMD 0.210–0.255 (0.220); PPW 0.243–0.285 (0.265); PSL 0.164–0.210 (0.182); PTW 0.174–0.210 (0.193); PW 0.467–0.550 (0.493); SL 0.728–0.863 (0.748); WL 1.012–1.175 (1.049). Indices: CI 77.4–84.0 (81.0); FI 116–127 (118); OI 26.8–28.9 (26.3); PI 128–140 (138); PSI 19.9–23.6 (22.2); SI 89.4–97.6 (90.7).

Head longer than broad in frontal view, widest anterior to eyes; posterior to eyes evenly narrowed toward slightly convex posterior margin. Antenna 12-segmented; scape exceeding posterior margin by more than its apical width; 3-segmented apical club poorly defined. Eyes with scattered very short setae arising between ommatidia; IOD 2.34–2.60 × EL; EL 0.96–1.15 × OMD. Mandibles coarsely longitudinally rugose. Clypeus with strong median carina and without flanking carinae between it and carina defining median lobe; lateral lobes each with several short longitudinal carinae. Malar area with several irregular coarse longitudinal rugae, some of which extend back mesad of eyes to posterolateral corners; others curve mesad over antennal fossa; interspaces shiny and weakly reticulate; frons and vertex shiny between fine sparse piligerous punctures. Dorsum with numerous long standing setae, longest exceeding one-half minimum eye diameter, setae slender and not appreciably flattened; venter with several shorter setae on each side.

Mesosoma slender, WL 2.07–2.17 × PW; in profile, dorsum sloping from pronotum to base of propodeal spines; propodeal spines about as long as distance between their bases. Mesosoma moderately shiny and weakly sculptured between coarse irregular longitudinal rugae; posterior face of propodeum with weak transverse rugae. Metafemur 4.77–5.15 times longer than wide in dorsal view. Entire length of dorsum with >40 long fully erect setae similar to those of frons, longest subequal to minimum eye diameter.

Petiole with short anterior peduncle; node thick-triangular in profile, posteriorly gently curved; subpetiolar tooth short, acute. Postpetiole node high and subrectangular in profile. Postpetiole node 1.28–1.40 times as wide as petiole node. Both nodes longitudinally rugose, petiole node with weakly reticulate interspaces at side, postpetiole weakly reticulate throughout. Setae on nodes numerous and similar to those of mesosomal dorsum.

Gaster in dorsal view 3.34–4.15 times wider than node of postpetiole; disc of first tergite moderately shiny and very finely reticulate throughout, between sparse fine piligerous punctures. Segments with numerous suberect setae similar to those of mesosoma.

Dark brown, gaster darker posteriorly.

Gyne and male unknown.

#### Material examined.

Holotype worker, U.S.A.: CALIFORNIA: Riverside Co.: Skinner Reservoir, 1360 m, 33.59°, -117.07°, 1–31.iii.1997 (T. Prentice) oak woodland, 1 worker (LACMENT299342) [LACM].

Paratypes. U.S.A.: CALIFORNIA: Riverside Co.: Skinner Reservoir, 1360 m, 33.59°, -117.07°, 1–31.iii.1997 (T. Prentice) oak woodland, 1 worker (LACMENT299339) [LACM], 1 worker (LACMENT299340) [LACM]; Riverside Co.: Skinner Reservoir, 1360 m, 33.59°, -117.07°, 1–30.ix.1997 (T. Prentice) oak woodland, 1 worker (LACMENT299341) [CASC], 1 worker (LACMENT299337) [MCZC].

Non-paratypic material examined. U.S.A.: CALIFORNIA: Orange Co.: Starr Ranch, 520 m, 33.6°, -117.55°, 2.iv.2001 (P. S. Ward, #PSW14325-4), ex midden of *Solenopsis xyloni*, coastal sage scrub, 1 worker (CASENT0339309) [UCDC]; Ventura Co.: Point Mugu State Park, 34.1367°, -118.9983°, 1–31.viii.2002 (F. Villalba) under chamise, 1 worker (CASENT0339314) [UCDC]; San Diego Co.: Miramar Naval Air Station, 32.87°, -117.06°, 20–30.viii.1997 (T. Prentice) oak woodland, 1 worker (LACMENT299338) [LACM]; San Diego Co.: Elliot Chaparral Reserve, 198 m, 32.8909°, -117.0975°, 1–30.vi.2005 (R. N. Fisher) 1 worker (CASENT0339323) [UCDC]; MEXICO: BAJA CALIFORNIA: Co.: Bahia San Quintín, 30.6°, -115.9°, 21.v.1952 (W. S. Creighton) 1 worker (LACMENT299335) [LACM]; 19.3 km S Guillermo Prieto, 27.7°, -113.3°, 7.iv.1982 (B. Phelps & M. Phelps) 1 worker (CASENT0339316) [UCDC]; 8.7 km NW Catavina, 27.79°, -114.78°, 21.ix.1981 (F. Andrew & D. Faulkner) 1 worker (LACMENT299336) [LACM].

#### Etymology.

The name is derived from the superficial resemblance to species of the unrelated genus *Myrmica*.

#### Remarks.

The large size, depressed mesosomal profile and long scapes will separate this from all other western *Temnothorax*. These features are shared with *Temnothorax paiute*, as is the presence of short setae on the eyes. The two differ, however, in the much more conspicuously and coarsely sculptured head and mesosoma of *Temnothorax myrmiciformis* (compare Figures 10 and 13).

The type series specimens were all collected in pitfall traps in grassy areas in oak woodland. The species has otherwise been collected in coastal sage scrub, chaparral, and Sonoran desert. The specimen collected by Creighton was taken near the beach at Bahia San Quintín, under a clump of *Mesembryanthemum*.

### Temnothorax neomexicanus

(W. M. Wheeler, 1903)

http://species-id.net/wiki/Temnothorax_neomexicanus

Figure 22

Leptothorax neomexicanus Wheeler 1903: 248, pl. 12, fig. 18. Five syntype workers, Manzanares, New Mexico [MCZC] [Images of MCZT_22783 syntype worker examined].
Leptothorax tricarinatus neomexicanus Wheeler; Creighton 1950: 273. Subspecies of *Leptothorax tricarinatus* Emery.
Leptothorax (Myrafant) tricarinatus neomexicanus Wheeler; Smith 1952: 100. First combination in *Leptothorax (Myrafant)*.
Leptothorax (Myrafant) neomexicanus Wheeler; Mackay 2000: 374. Revived status as species.
Temnothorax neomexicanus (Wheeler); Bolton 2003: 271. First combination in *Temnothorax*.

#### Material examined.

8 series from California, 2 series from Nevada, 4 series from Arizona [MLBC, MMPC, UCDC].

#### Remarks.

See Mackay (2000) for a full description of this species.

### Temnothorax nevadensis

(Wheeler, 1903)

http://species-id.net/wiki/Temnothorax_nevadensis

Figures 21 –22

Leptothorax nevadensis Wheeler 1903: 252. Two syntype workers, two syntype males, one dealate queen, Kings Cañon, Ormsby Co., Nevada (C. F. Baker) [MCZC] [Images of MCZ_21033 syntype worker examined]
Leptothorax melanderi Wheeler 1909: 81. Holotype worker, Moscow Mountain, Idaho (A. L. Melander) [AMNH] [Not examined]. Synonymy by Mackay 2000: 376.
Leptothorax eldoradensis Wheeler 1915: 414. Two syntype workers, Mt. Wilson, California (J. C. Bradley) [MCZC] [Not examined]. Synonymy by Mackay 2000: 376; Confirmed by Ward 2005: 16.
Leptothorax lindae Mackay 2000: 356. Holotype worker, Wolverton Campground, Sequoia Natl. Park, Tulare Co., California (W. & L. Mackay) [MCZC] [Not examined]. Paratype workers (same data) in LACM [Not examined]. Synonymy by Ward 2005: 16.
Leptothorax maryanae Mackay 2000: 364. Holotype worker, 4 mi N Fawnskin, San Bernardino Mnts, California (B. & E. Mackay) [MCZC] [Not examined]. Synonymy by Ward 2005: 17.
Temnothorax lindae (Mackay); Bolton 2003: 271. First combination in *Temnothorax*.
Temnothorax maryanae (Mackay); Bolton 2003: 271. First combination in *Temnothorax*.
Temnothorax nevadensis (Wheeler); Bolton 2003: 271. First combination in *Temnothorax*.

#### Material examined.

132 series from California, 12 series from Nevada, 3 series from Utah, 6 series from Oregon, 3 series from Washington, 1 series from Montana [MLBC, MMPC, PSWC, UCDC].

#### Remarks.

See Ward (2005) for a discussion of this species.

### Temnothorax nitens

(Emery, 1895)

http://species-id.net/wiki/Temnothorax_nitens

Figure 21 – 23

Leptothorax nitens Emery 1895: 322.Holotype worker, American Fork Canyon, Utah [USNM] [Images of USNMENT00531621 holotype worker examined]
Leptothorax nitens var. *mariposa*Wheeler 1917: 507. Nine syntype workers, Camp Curry, Yosemite, California [LACM, MCZC] [Images of CASENT0105618 and MCZ_8677 syntype workers examined]. Synonymy by Ward 2005: 17.
Leptothorax mariposa Wheeler; Cole 1958b: 536. Raised to species.
Leptothorax melinus Mackay 2000: 368. Holotype worker and 12 paratype workers, Beartrap Cyn., Socorro Co., New Mexico, 2286 m (W. Mackay #16889) [MCZC, CASC, WPMC, LACM, MCZC, MZSP, USNM] [Images of CASENT0105864 paratype examined]. Synonymy by Ward 2005: 17.
Temnothorax mariposa (Wheeler); Bolton 2003: 271. First combination in *Temnothorax*.
Temnothorax melinus (Mackay); Bolton 2003: 271. First combination in *Temnothorax*.
Temnothorax nitens (Emery); Bolton 2003: 271. First combination in *Temnothorax*.
Temnothorax nitens (Emery); Ward 2005: 17-19. Senior synonym of *Temnothorax mariposa* and *Temnothorax melinus*.

#### Material examined.

107 series from California, 24 series from Nevada, and 7 series from Arizona [MPPC, PSWC, UCDC].

#### Remarks.

See Ward (2005) for a discussion of this species.

### Temnothorax nuwuvi sp. n.

http://zoobank.org/E3E0C58D-0B96-4971-82B5-E544481475DD

http://species-id.net/wiki/Temnothorax_nuwuvi

**Figure 11.**
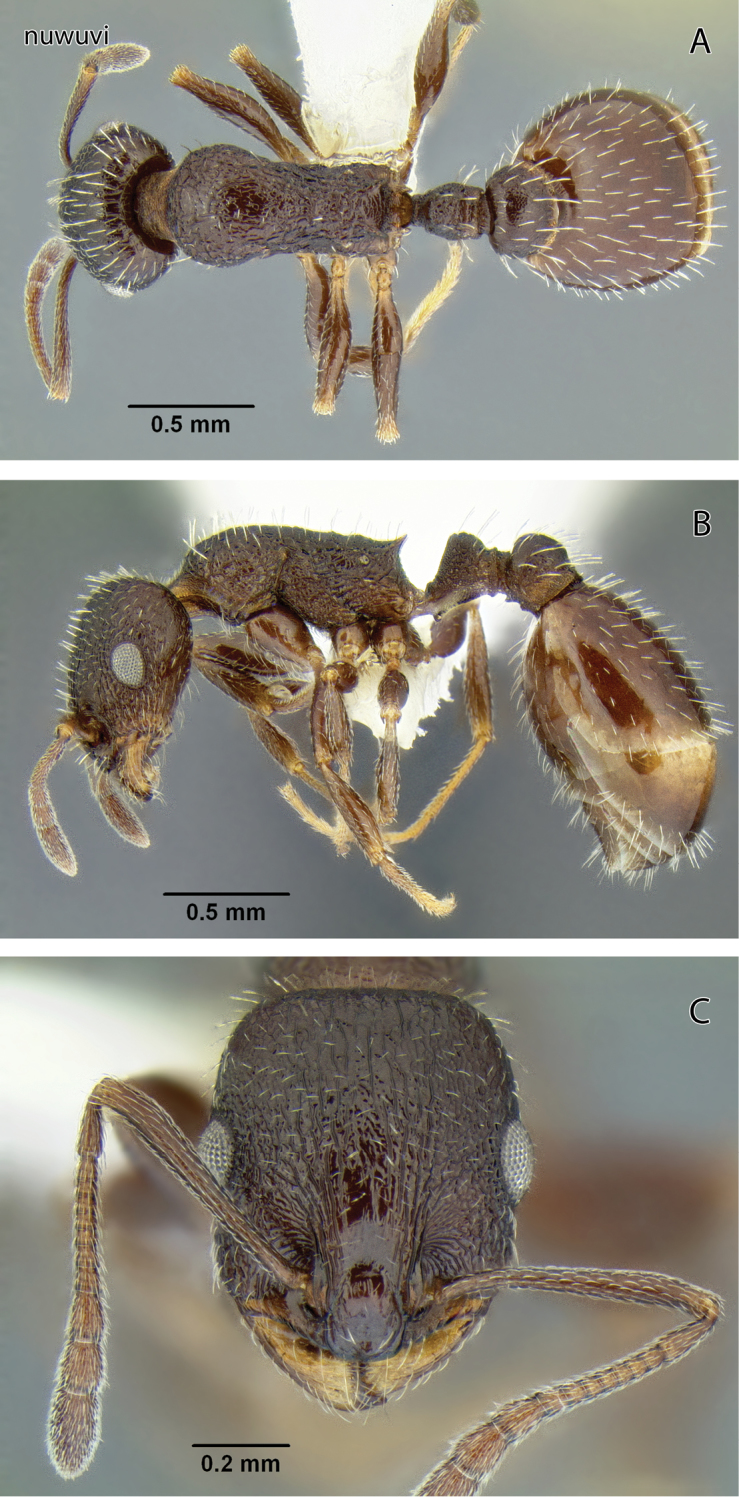
*Temnothorax nuwuvi* sp. n., worker (LACMENT299376) **A** Body in dorsal view **B** Body in lateral view **C** Head in full-face view.

**Figure 12.**
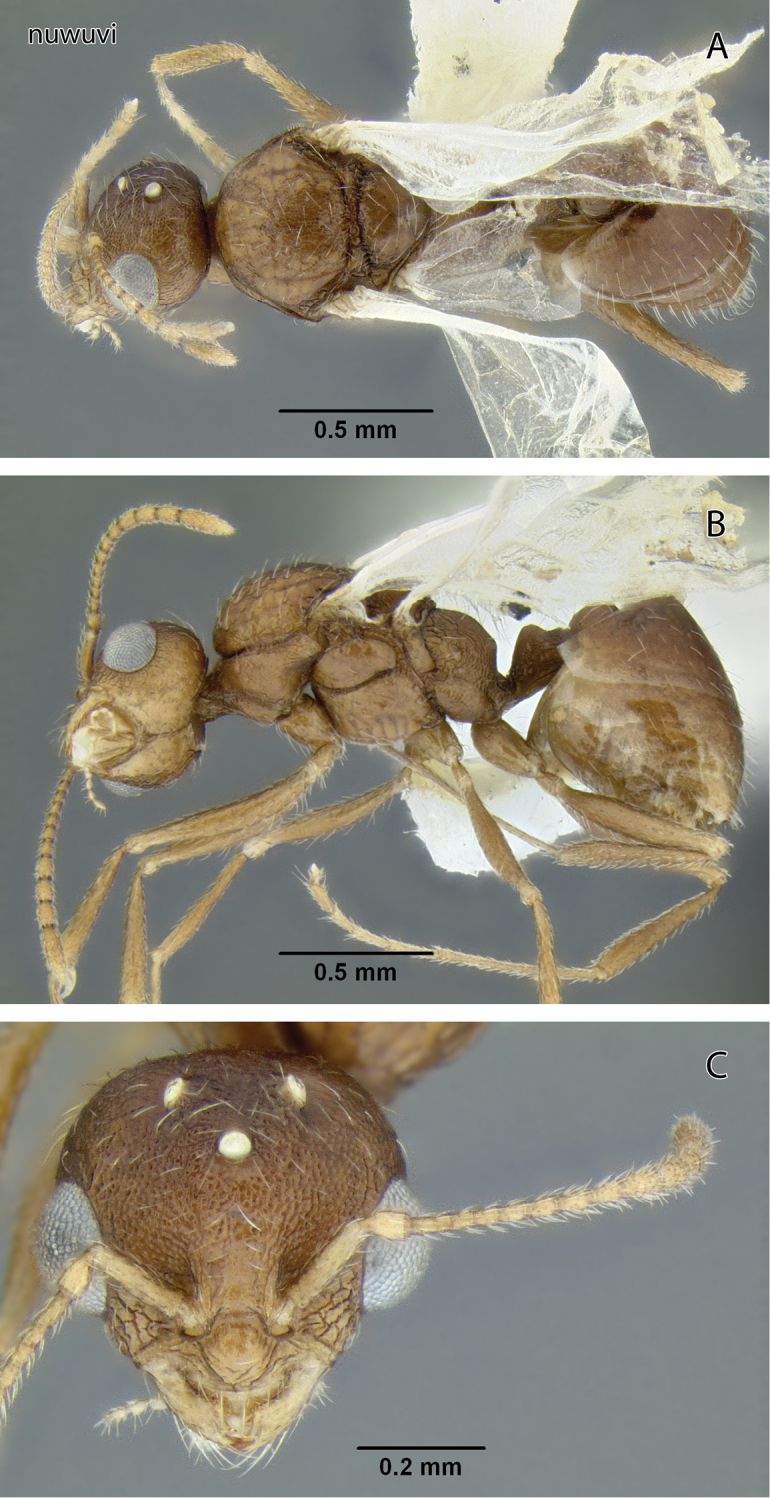
*Temnothorax nuwuvi* sp. n., male (LACMENT299382) **A** Body in dorsal view **B** Body in lateral view **C** Head in full-face view.

Figures 11 –12 , 19 , 23 –24

#### Diagnosis of worker.

Head sculptured with longitudinal rugae anastomosing into reticulation on sides; petiole node robust and subtriangular in profile with rounded summit; propodeal spines acute and relatively short; dark reddish brown.

#### Description.

Worker measurements (mm) (40 measured): EL 0.163–0.196 (0.180); HFL 0.540–0.650 (0.583); HFW 0.098–0.147 (0.127); HL 0.646–0.771 (0.701); HW 0.532–0.662 (0.586); IOD 0.459–0.558 (0.504); OMD 0.141–0.184 (0.159); PPW 0.302–0.367 (0.328); PSL 0.066–0.118 (0.093); PTW 0.179–0.228 (0.200); PW 0.387–0.502 (0.430); SL 0.519–0.610 (0.554); WL 0.793–0.977 (0.867). Indices: CI 78.7–96.8 (83.6); FI 84.0–107.9 (99.5); OI 23.5–27.8 (25.7); PI 154–176 (164); PSI 9.9–16.9 (13.2); SI 74.6–84.8 (79.1).

Head longer than broad in frontal view, lateral margins very weakly convex, posterior margin flat to very weakly convex. Antennal scape just outreaching posterior margin; apical club distinctly 3-segmented. IOD 2.53–3.02 × EL; EL 0.99–1.27 × OMD. Mandible finely longitudinally rugose. Clypeus with 1 or 2 short fine carinae on either side of median carina. Dorsum of head with irregular longitudinal rugae, interspaces from mostly smooth between rugae along dorsum midline to contiguously reticulate laterally; malar area with several short fine longitudinal rugae, interspaces dull and sharply reticulate; posterolateral angles of head moderately shiny and weakly reticulate. Dorsum of head with relatively dense, blunt setae.

Mesosoma slender, WL 1.78–2.11 times longer than PW; mesosomal dorsum weakly convex in profile, sloping down to sharp propodeal spines. Mesosomal dorsum moderately rugose with corrugated interspaces and the corrugation faintest medially on pronotum; side of pronotum coarsely rugose, interspaces inconsistently corrugated; posterior face of propodeum dull and reticulate. Metafemur 4.26–5.38 times longer than wide in dorsal view. Entire length of dorsum with 20+ blunt setae that are distinctly longer than those of frons.

Petiole with only very short anterior peduncle; node robust and subtriangular, no higher than thick, summit flattened; subpetiolar tooth short and acute. Postpetiole profile low; in dorsal view node 1.54–1.76 times width of petiole node. Petiole coarsely rugose laterally and dorsally with interspaces corrugated and anterior face reticulate; postpetiole with much weaker rugosity laterally and posteriorly, with semi–regular corrugation transitioning to fading reticulum dorsally; dorsum of postpetiole can be relatively smooth, but traces of reticulum can be seen in most specimens. Petiole with 6–8 setae similar to those of mesosoma and postpetiole with 14–16 setae.

Gaster in dorsal view 1.93–2.36 times wider than postpetiole node; surface of first tergite smooth and shiny between sparse piligerous punctures; surface with sparse suberect flattened setae similar to those of mesosoma and scattered finer prostrate pubescence.

Head and body dark brown.

Gyne unknown.

Male measurements (mm) (2 measured): EL 0.221–0.234 (0.228); HFL 0.673–0.685 (0.679); HFW 0.085–0.094 (0.090); HL 0.422–0.485 (0.454); HW 0.502–0.503 (0.502); IOD 0.377–0.397 (0.387); OMD 0.073–0.081 (0.077); PPW 0.231–0.236 (0.233); PSL N/A; PTW 0.147–0.161 (0.154); PW 0.519–0.623 (0.571); SL 0.164–0.178 (0.171); WL 0.936–0.973 (0.955). Indices: CI 104–119 (111); FI 134–136 (135); OI 45.6–55.5 (50.5); PI 147–157 (152); PSI N/A; SI 33.8–42.2 (38.0).

Head broader than long, margins behind eyes slightly convergent and broadly rounded into slightly convex posterior margin. Eyes large and strongly bulging; IOD 1.70–1.71 x EL; OMD very short, about equal to transverse diameter of anterior ocellus; interocellar distance 3.30–3.50 and ocellocular distance 3.60–4.10 times diameter of anterior ocellus. Antennae 13-segmented. Scape less than half of IOD. Head dull, mostly reticulate with few longitudinal rugae near frontal carinae and ocelli. Dorsum of head with about a dozen erect, relatively long and pointed setae, those setae are located mostly on posterior half of head dorsum, and on vertexal margin behind ocelli.

Mesosoma robust, WL 1.56–1.80 × PW. Sculpture pronounced. Entire pronotum reticulate. Most of mesoscutal dorsum shagreened, and mesoscutellum medially smooth. Mesoscutum with >30 suberect short, slender setae; four pairs of longer setae submedially on metanotum. Small propodeal tubercles present. Entire propodeum reticulate with weak rugosities near the tubercles.

Summit of petiole node low, angular in profile; subpetiolar process absent.

Body brown with gaster and appendages lighter.

#### Material examined.

Holotype worker, U.S.A.: NEVADA: Nye Co.: Mount Jefferson, 2895 m, 38.7°, -116.9°, 7.viii.1976 (G. C. Wheeler & J. Wheeler) (LACMENT299376) [LACM].

Paratypes. Same data as holotype paratype, 2 workers (LACMENT299381) [MCZC], 2 workers (LACMENT299380) [USNM], 2 workers (LACMENT299379) [LACM], 2 workers (LACMENT299378) [LACM], 2 workers (LACMENT299377) [LACM], 3 workers (LACMENT299375) [LACM], 2 workers (LACMENT299386) [UCDC].

Non-paratypic material examined. U.S.A.: NEVADA: Nye Co.: Mount Jefferson, south side, 3050 m, 38.7°, -116.9°, 7.viii.1976 (G. C. Wheeler & J. Wheeler) 1 worker, 1 male (LACMENT299384) [LACM], 2 workers (LACMENT299383) [LACM], 2 workers, 1 male (LACMENT299382) [LACM]; Nye Co.: Manhattan, 2255 m, 38.5°, -117.1°, 7.viii.1976 (G. C. Wheeler & J. Wheeler) 2 workers (LACMENT299395) [UCDC], 2 workers (LACMENT299393) [LACM], 2 workers (LACMENT299392) [LACM], 2 workers (LACMENT299391) [LACM], 2 workers (LACMENT299390) [LACM], 2 workers (LACMENT299389) [LACM], 2 workers (LACMENT299388) [LACM], 2 workers (LACMENT299387) [LACM], 2 workers (LACMENT299394) [LACM], 3 workers (LACMENT299385) [LACM], 1 worker (CASENT0172608) [LACM].

#### Etymology.

The name “Nuwuvi” is the Southern Paiute name for themselves; it means “the People” or the true men.

#### Remarks.

The combination of brown body color with relatively coarse head sculpture, weakly sculptured postpetiole dorsum, non-acute petiole node (Figure 19F) and postpetiole more than 1.5 × as wide as long in dorsal view will serve to distinguish this species from other *Temnothorax* occurring in the area. With regard to color and sculpture, *Temnothorax nuwuvi* is similar to *Temnothorax nevadensis*. The latter is a very variable species, but the combination of less robust petiolar node, postpetiole relatively more narrow, and longer propodeal spines in *Temnothorax nevadensis* (PI 129–147, PSI 16–26 in *nevadensis* vs. PI 154–176, PSI 10–17 in *nuwuvi*) (Figures 11B, 21D) will serve to distinguish the two.

### Temnothorax oxynodis

(Mackay, 2000)

http://species-id.net/wiki/Temnothorax_oxynodis

Figure 22

Leptothorax (Myrafant) oxynodis Mackay, 2000: 385. Holotype worker, near Big Basin, Santa Cruz Co., California. U.S.A. [CASC] [Images of CASTYPE17706 holotype worker examined].
Temnothorax oxynodis (Mackay); Bolton 2003: 272. First combination in *Temnothorax*.

#### Material examined.

3 series from California [PSWC, UCDC].

#### Remarks.

See Mackay (2000) for a full description of this species.

### Temnothorax paiute sp. n.

http://zoobank.org/F6A83AFD-226F-4CB0-BAAD-F1C49D3DE49B

http://species-id.net/wiki/Temnothorax_paiute

**Figure 13.**
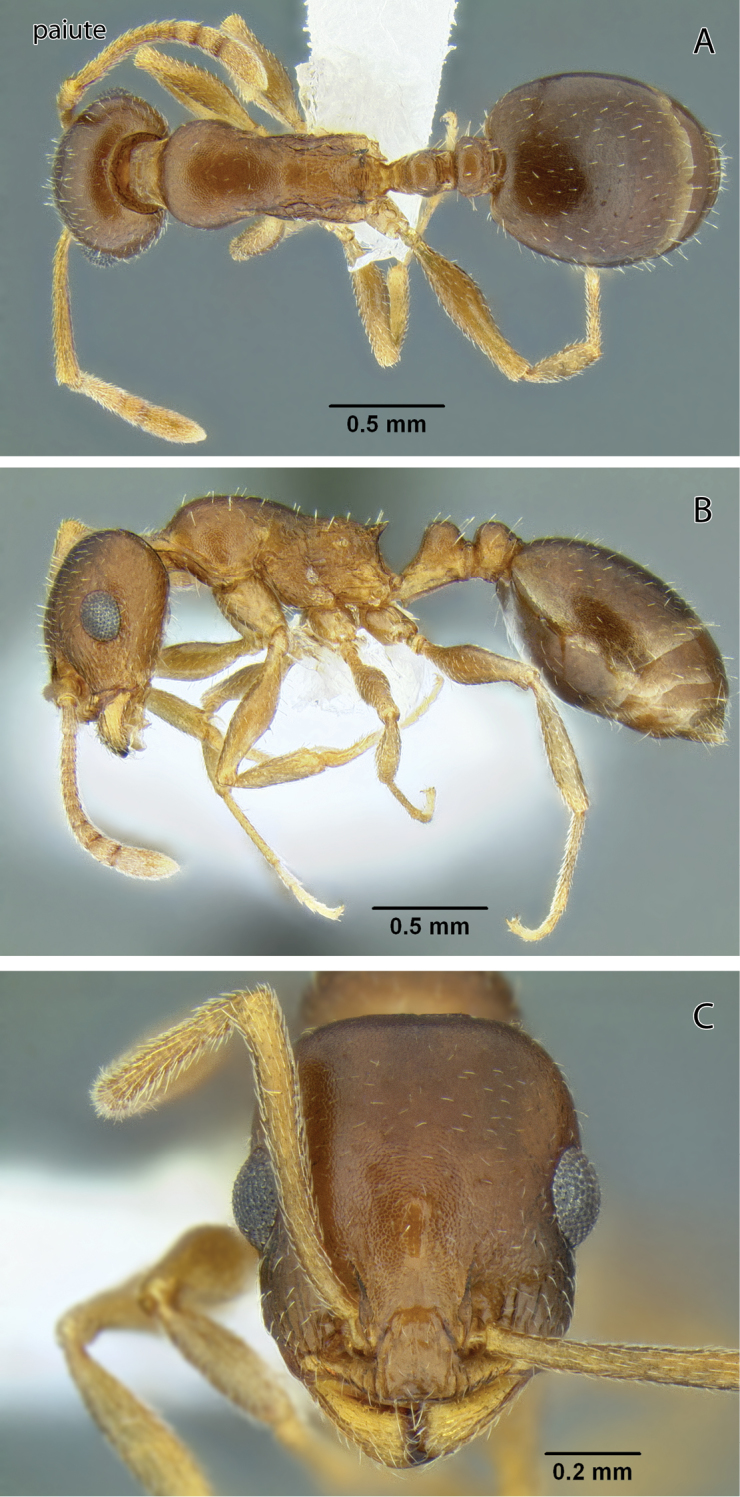
*Temnothorax paiute* sp. n., worker (CASENT0005932) **A** Body in dorsal view **B** Body in lateral view **C** Head in full-face view.

Figures 13 , 20 , 24

Temnothorax sp. CA-08; Ward 2005: 68.

#### Diagnosis of worker.

Mesonotum distinctly sloping down to metanotal area and base of propodeum slightly convex; first gastral tergum slightly shiny and very finely sculptured throughout; side of head behind eyes with no more than 1 or 2 short fine setae.

#### Description.

Worker measurements (mm) (4 measured). EL 0.204–0.224 (0.216); HFL 0.680–0.738 (0.720); HFW 0.145–0.170 (0.155); HL 0.780–0.828 (0.806); HW 0.642–0.695 (0.671); IOD 0.531–0.583 (0.566); OMD 0.187–0.218 (0.201); PPW 0.224–0.270 (0.248); PSL 0.125–0.133 (0.128); PTW 0.172–0.212 (0.193); PW 0.440–0.491 (0.472); SL 0.706–0.741 (0.724); WL 0.964–1.028 (0.999). Indices: CI 82.3–83.9 (83.2); FI 106–111 (107); OI 26.2–27.2 (26.8); PI 125–131 (128); PSI 15.1–16.9 (15.9); SI 87.0–92.5 (89.9).

Head longer than broad in frontal view; posterior margin transverse and lateral margins essentially parallel. Antenna 12-segmented; scape long and exceeding posterior margin by more than its apical width; antennal club distinct, 3-segmented. Eye large and moderately bulging, IOD about 2.54–2.71 × EL; with a few widely scattered very short setae arising between ommatidia; EL 0.98–1.17 × OMD. Mandibles coarsely longitudinally rugose. Head slightly shiny and conspicuously finely reticulate and with widely scattered obscure minute punctures; interrugal surfaces of clypeus smooth and shiny; malar area with several short obscure rugulae. Sparse short yellowish erect to suberect setae on front of head; more abundant suberect to erect setae on hypostomal area.

Mesosoma slender, WL 2.08–2.19 times longer than PW; in profile, posterior portion of mesonotum sloping down to metanotal impression, behind which dorsal face of propodeum is slightly raised and weakly convex. Propodeal spines present and well–developed: PSI 15.1–16.9; stout and shorter than infraspinal distance in largest specimens (California), more slender and longer than infraspinal distance in smaller (Nevada) specimens. Entire pronotum and mesosomal dorsum slightly shiny and finely reticulate, without rugae; mesepisternum and side of propodeum with conspicuous more or less longitudinal rugae, metapleural suture with about 6 coarse cross–rugae. Metafemur slender, 4.29–4.93 times longer than wide in dorsal view. Dorsum, including propodeum, with sparse yellowish slender erect setae.

Petiole node high in profile, summit broadly rounded; acute subpetiolar tooth present; summit of postpetiole rounded in profile; in dorsal view quadrate to slightly trapezoidal. Both nodes slightly shiny and finely reticulate, without rugae and with sparse yellowish slender erect setae.

Gaster, in dorsal view, 3.12–3.54 times broader than width of postpetiole; slightly shiny and first tergum finely reticulate throughout; sparse yellowish slender erect setae evenly distributed on first tergum; first tergum also with widely scattered finer subappressed setae.

Color of body medium brown with gaster darker.

Gyne and male unknown.

#### Material examined.

Holotype worker, U.S.A.: CALIFORNIA: Inyo Co.: Lone Pine, 1310 m, 36.6°, -118.1°, 7.iv.1959 (W. S. Creighton) (CASENT0005932) [LACM].

Paratypes. 1 worker, same data as holotype (CASENT0005932) [LACM].

Non-paratypic material examined. U.S.A.: NEVADA: Nye Co.: Nevada Test Site, 36.7°, -116°, 12.ii.1962 (A. C. Cole) 1 worker (LACMENT299343) [LACM]; Nye Co.: Nevada Test Site, 36.7°, -116°, 14.xi.1961 (A. C. Cole) on *Ephedra nevadensis*, 1 worker (LACMENT299344) [LACM].

#### Etymology.

This species is named for the peoples of the Paiute Nation and is to be treated as a noun in apposition.

#### Remarks.

The two specimens from Nevada have proportionately slightly longer scapes than those of the Lone Pine specimens (SI 90.5–92.5 vs. 87.0–89.5), but otherwise share the unique features cited in the diagnosis. Although *Temnothorax paiute* unquestionably belongs among the species placed in the former subgenus *Myrafant*, characterized within our fauna by the presence of a median longitudinal clypeal carina and the absence of the metanotal suture across the dorsum, it does not appear to belong to any of the previously recognized species groups. Also see above under *Temnothorax myrmiciformis*.

Cole’s label data indicate that a specimen has been collected on *Ephedra nevadensis* but otherwise nothing is known about this species’ biology.

### Temnothorax pseudandrei sp. n.

http://zoobank.org/5A46B981-1AD6-40A4-9EF3-75B1A9D06C80

http://species-id.net/wiki/Temnothorax_pseudandrei

**Figure 14.**
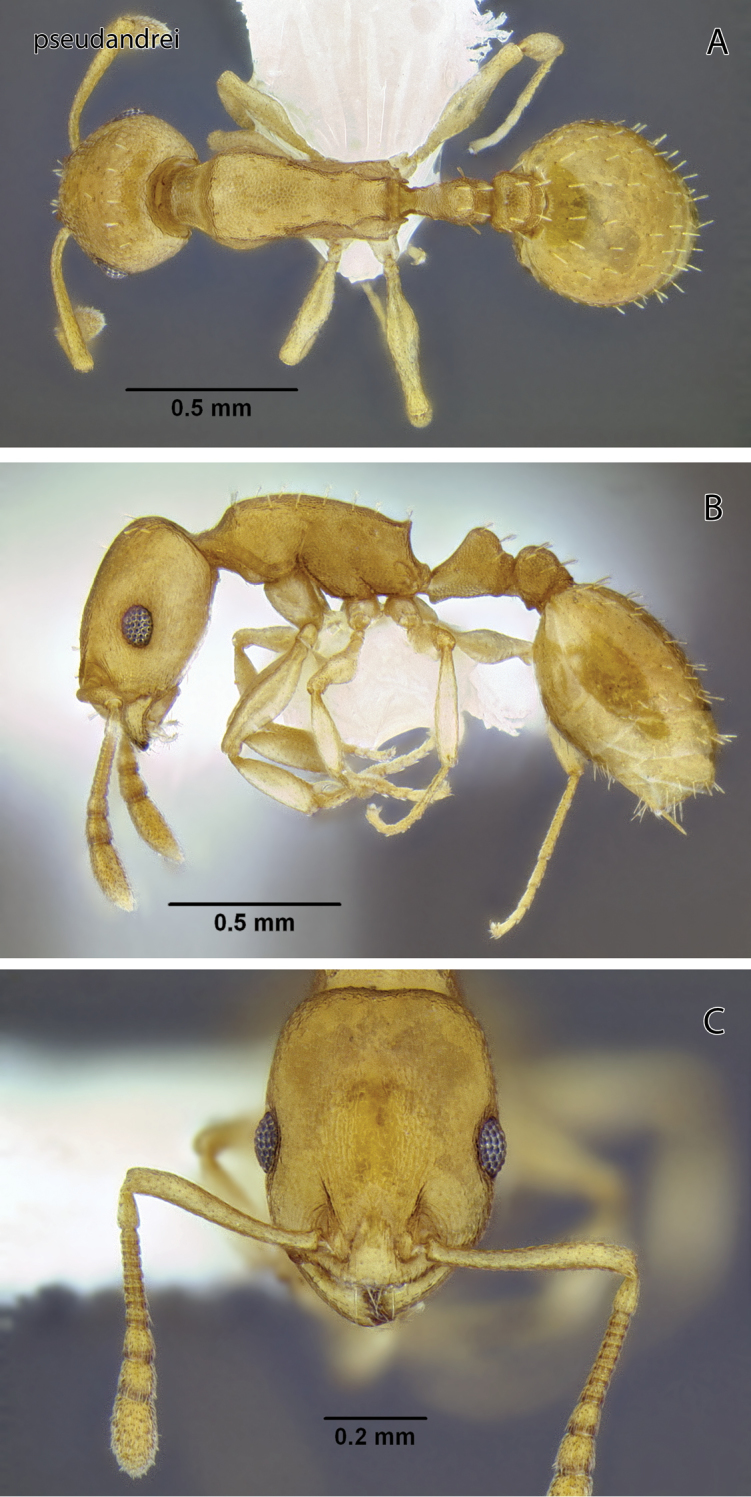
*Temnothorax pseudandrei* sp. n., worker (CASENT0005693) **A** Body in dorsal view **B** Body in lateral view **C** Head in full-face view.

**Figure 15.**
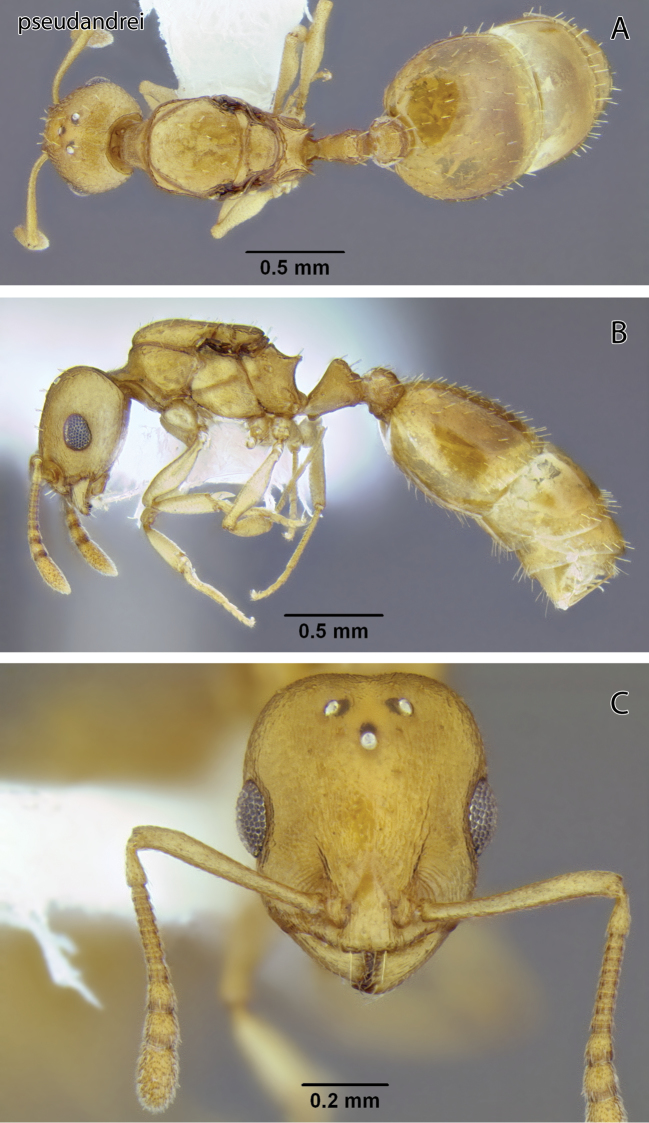
*Temnothorax pseudandrei* sp. n., dealate gyne (CASENT0339311) **A** Body in dorsal view **B** Body in lateral view **C** Head in full-face view.

**Figure 16.**
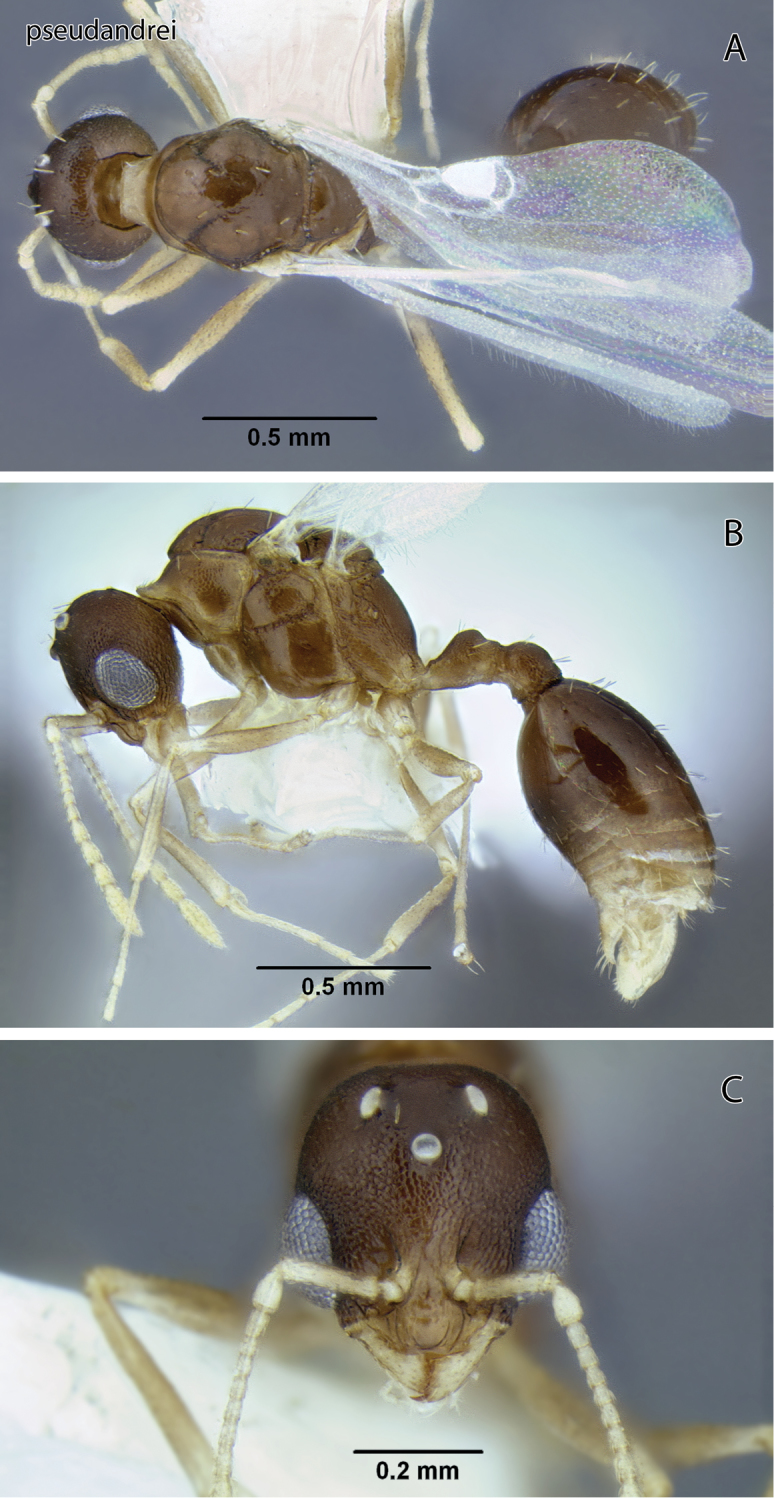
*Temnothorax pseudandrei* sp. n., male (CASENT0005693) **A** Body in dorsal view **B** Body in lateral view **C** Head in full-face view.

Figures 14 – 16 , 20 , 24 –25

Temnothorax sp. CA-04; Ward 2005: 68.

#### Diagnosis of worker.

Mesosoma, petiole and postpetiole dull and contiguously punctate; head shinier, punctures larger, shallower and distinctly shiny within, without fine rugae except above frontal lobes and around antennal fossa; subpetiolar process short and broad.

#### Description.

Worker measurements (mm) (12 measured): EL 0.121–0.152 (0.139); HFL 0.408–0.488 (0.442); HFW 0.095–0.109 (0.102); HL 0.577–0.648 (0.619); HW 0.454–0.514 (0.489); IOD 0.390–0.430 (0.413); OMD 0.134–0.155 (0.144); PPW 0.179–0.196 (0.188); PSL 0.049–0.080 (0.066); PTW 0.130–0.153 (0.145); PW 0.296–0.345 (0.324); SL 0.394–0.457 (0.432); WL 0.627–0.715 (0.675). Indices: CI 77.5–80.7 (79.1); FI 86.4–96.4 (90.4); OI 21.0–23.7 (22.4); PI 123–138 (130); PSI 7.73–12.6 (10.7); SI 68.3–71.0 (69.8).

Head longer than broad in frontal view; posterior margin mostly transverse but with small median impression; lateral margins very weakly convex and slightly convergent behind eyes. Antenna 12-segmented; scape short of posterior margin by slightly more than its apical width; apical club distinctly 3-segmented. Eye small and slightly bulging in frontal view; IOD 2.77–3.22 × EL; EL 0.89–1.05 × OMD. Most of head slightly shiny, with reticulation shiny within; midline of frons shinier, less strongly sculptured and with indistinct median smooth line; very fine rugae curving around antennal fossa; additional very fine rugae extending back from frontal lobes but ending well below vertex; median and submedian clypeal carinae weak. Dorsum of head with scattered very short to short (one vertex) flattened yellowish setae.

Mesosoma slender, WL 2.03–2.17 × PW in dorsal view; dorsal profile slightly convex. Propodeal spines short and acute, about half as long as distance between their bases. Entire mesosoma dull and reticulate, without rugulae or reticulae. Metafemur 3.93–4.69 times as long as wide in dorsal view. Dorsum with 16–18 short erect flattened yellowish setae.

Petiole node blunt and rounded in profile; subpetiolar process short and obtuse in profile; postpetiole node low and broadly rounded. Sculpture of both nodes similar to that of mesosoma.

Gaster in dorsal view about 2.87–3.28 times wider than postpetiole; first tergum smooth and shiny between scattered fine piligerous punctures; setae short and flattened.

Head and body uniformly pale yellow reddish yellow; gaster slightly darker or no darker.

Gyne: measurements (mm) (1 measured): EL 0.202; HFL 0.530; HFW 0.107; HL 0.672; HW 0.572; IOD 0.468; OMD 0.131; PPW 0.246; PSL 0.115; PTW 0.187; PW 0.558; SL 0.472; WL 0.995. Indices: CI 85.1; FI 92.7; OI 30.1; PI 132; PSI 17.1; SI 70.2.

Head longer than broad, margins approximately parallel in frontal view and broadly rounded into transverse posterior margin. Antennal scape extending back to level of anterior ocellus. Eye large and moderately convex, IOD 2.32 × EL in frontal view; EL 1.54 × OMD. Sculpture and pilosity about as in worker.

Mesosoma slender, WL 1.78 × PW. Mesonotum flat in profile. Propodeal spines thick at base, about half as long as infraspinal distance. Mesonotum weakly shiny and mostly reticulate, with some indistinct punctation behind; side of pronotum duller and distinctly reticulate; mesepisternum shinier but reticulate throughout; propodeum dull, reticulate. Pilosity suberect to erect, short (less than 0.05 mm) and sparse, distinctly flattened and blunt-tipped.

Remainder as described for worker; gaster 3.40 times as wide as postpetiole.

Male measurements (mm) (7 measured): EL 0.184–0.204 (0.195); HFL 0.517–0.608 (0.555); HFW 0.061–0.070 (0.065); HL 0.427–0.445 (0.439); HW 0.387–0.410 (0.403); IOD 0.253–0.277 (0.267); OMD 0.036–0.054 (0.046); PPW 0.162–0.222 (0.182); PSL N/A; PTW 0.116–0.131 (0.121); PW 0.358–0.483 (0.409); SL 0.163–0.176 (0.169); WL 0.798–0.888 (0.844). Indices: CI 90.6–92.6 (91.7); FI 131–148 (138); OI 42.9–46 (44.4); PI 133–190 (150); PSI N/A; SI 37.3–39.6 (38.5).

Head longer than broad, margins behind eyes slightly convergent and broadly rounded into transverse posterior margin. Eyes large and strongly bulging; IOD 1.27–1.44 × EL; OMD very short, about equal to transverse diameter of anterior ocellus (EL 3.61–5.67 times OMD); interocellar distance 2.00–2.30 and ocellocular distance 3.00–3.30 × diameter of anterior ocellus. Antennae 13-segmented. Scape distinctly shorter than IOD. Head moderately shiny, weakly and shallowly reticulate and with variable irregular smoother areas. Dorsum of head without erect setae other than three on each side in ocellar area, those setae very short and flattened.

Mesosoma robust, WL 1.84–2.35 × PW. Propodeal spines absent. Sculpture fine and weak, mesoscutal dorsum smooth and shiny. Mesoscutum with 3–4 pairs of suberect short, slender setae; a single pair of longer setae submedially on metanotum.

Summit of petiole node low, angular in profile; subpetiolar process absent.

Mesosoma and petiolar segments dark brown brownish yellow; appendages whitish head and gaster reddish brown.

#### Material examined.

Holotype worker. U.S.A.: ARIZONA: Pinal Co.: Kearney near Gila River, 560 m, 33.050°, -110.915°, 24.vi.1996 (R. A. Johnson, #AZ 882), nest in bark of *Populus fremontii* (CASENT0339311) [UCDC].

Paratypes. Same data as holotype, 1 dealate gyne, 1 worker (CASENT0339311) [UCDC], 2 workers, 1 male (CASENT0005693) [USNM], 1 worker, 1 male (LACMENT299370) [LACM], 2 workers (LACMENT299371) [LACM], 2 workers (LACMENT299372) [LACM], 1 worker (LACMENT299373) [MCZC].

Non-paratypic material examined. U.S.A.: CALIFORNIA: Imperial Co.: Bard, 32.79°, -114.55°, 17.iii.1964 (G. Beevor) under bark of *Chilopsis linearis*, 1 worker (LACMENT299359) [LACM], 1 worker (LACMENT299360) [LACM], 1 worker (LACMENT299361) [LACM], 1 worker (LACMENT299374) [LACM]; Inyo Co.: Resting Springs, 35.87°, -116.16°, 29–30.v.1955 (J. N. Belkin et al.) presumably at lights, 1 dealate gyne (LACMENT299362) [LACM], 1 male (LACMENT299363) [LACM], 1 male (LACMENT299364) [LACM], 1 male (LACMENT299365) [LACM], 1 male (LACMENT299366) [LACM], 1 male (LACMENT299367) [LACM], 1 male (LACMENT299368) [LACM].

#### Etymology.

From Greek, pseudos (false) + andrei, for the close resemblance of this species to *Temnothorax andrei*.

#### Remarks.

As the name suggests, *Temnothorax pseudandrei* is similar to, and may be confused with, *Temnothorax andrei*, a common ground-dwelling species in California and neighboring states. Workers of *Temnothorax pseudandrei* differ by the short, broad subpetiolar process and the less robust, more acute petiolar node (Figures 20B, 21A). The worker of *Temnothorax pseudandrei* lacks erect setae on the underside of the head and the gyne has a single pair of very short flattened setae a short distance behind the oral cavity. Fine erect setae are present on the underside of the head in both workers and gynes of *Temnothorax andrei*. Additionally, the erect setae on the cephalic and mesosomal dorsa are shorter and less numerous.

Males of the two species are similar but differ in the length of the few setae present on the upper frons and vertex. In *Temnothorax andrei* there are about 10 setae, including 2 along the lateral margins and all are distinctly longer than the maximum diameter of the anterior ocellus. A few setae are also present on the underside of the head. Males of *Temnothorax pseudandrei* have about 6 setae on the vertex, none on the frons none along the lateral margins and none on the underside of the head. Those setae present on the vertex are only slightly longer than the greatest diameter of the anterior ocellus.

Little is known of the ecology of this ant other than that it is an arboreal species. As noted above, the Bard specimens were taken from under the bark of desert willow (*Chilopsis linearis*) in habitat that is now a much fragmented riverine gallery forest. The habitat for the Arizona specimens is similar, but in this instance the specimens were nesting in the bark of Fremont cottonwood (*Populus fremontii*). No data are available for the Resting Springs specimens and they were presumably taken at lights at night. That habitat is not riverine, but originally there was a flowing spring and a substantial stand of Fremont cottonwood. The site has been commercially developed and most of the cottonwood trees are no longer there.

### Temnothorax quasimodo sp. n.

http://zoobank.org/CF5B1050-72F1-4D42-9130-B1481885DF23

http://species-id.net/wiki/Temnothorax_quasimodo

Figures 17 , 24

Temnothorax sp. CA-05; Ward 2005: 68.

#### Diagnosis of worker.

Mesosoma short and deep in profile, dorsum distinctly convex; propodeum without spines but with blunt obtuse angles.

#### Description.

Worker measurements (mm) (1 measured). EL 0.090; HFL 0.334; HFW 0.102; HL 0.529; HW 0.416; IOD 0.364; OMD 0.123; PPW 0.250; PSL N/A; PTW 0.193; PW 0.298; SL 0.339; WL 0.587. Indices: CI 78.6; FI 80.3; OI 17.0; PI 130; PSI N/A; SI 64.1.

Head longer than broad in frontal view; posterior margin transverse and weakly concave in middle; lateral margins weakly curved and slightly convergent toward mandibular bases. Antenna 12-segmented; scape short and ending well below posterior margin; apical club distinctly three-segmented. Eye small, IOD 4.04 × EL; EL 0.73 times OMD. Mandible moderately coarsely rugulose (rugulae finer than in *Temnothorax andrei* Emery). Head moderately shiny and with narrow shiny median line; surface mostly very finely lineolate (“striate”) and with dense (especially anteriorly) minute shallow punctures becoming sparser and less distinct posteriorly and with scattered coarser piligerous punctures; antennal fossa and malar area only slightly shiny and roughened between well-separated fine rugulae (straight on malar area, curving mesad over fossa). Dorsum of head with widely scattered short, erect to suberect yellowish setae, the setae stiff and slightly flattened; similar but longer setae beneath.

Mesosoma stout, WL 1.97 times PW; mesosomal profile deep, distance from dorsal mesonotal margin to base of mesocoxa 0.50 times WL; mesosomal dorsum distinctly convex in profile. Propodeal spines reduced to blunt obtuse angles in profile. Mesosoma opaque and reticulate, except infraspinal face of propodeum less strongly sculptured and shinier. Dorsum with about 20 sparse slightly flattened yellowish setae that are longer than those of head. Metafemur robust, 3.27 times longer than wide in dorsal view.

Petiole lacking anterior peduncle; node robustly triangular in profile with rounded summit; subpetiolar process consisting of a distinct thin flange that is acute anteriorly. Postpetiolar node profile high and rounded and in dorsal view trapezoidal with pronounced anterior corners and 1.30 times width of petiolar node. Sculpture and pilosity of both nodes similar to those of mesosoma.

Gaster in dorsal view 2.26 times wider than node of postpetiole; disc of first tergum smooth and shiny with scattered minute piligerous punctures; setae fine and yellowish, slightly flattened; remaining segments with similar setae.

Color of body and appendages uniformly yellow.

Gyne and male unknown.

#### Material examined.

Holotype worker, U.S.A.: CALIFORNIA: Solano Co.: Cold Canyon, 19 km NNW Vacaville, 300m, 38.5°, -122.1°, 25.ii.1990 (P. S. Ward, #10562-3) ex sifted litter (leaf mold, rotten wood), chaparral (CASENT0005694) [UCDC].

#### Etymology.

*Temnothorax quasimodo* is named for the Victor Hugo character in his novel The Hunchback of Notre Dame.

#### Remarks.

The short, deep mesosoma with distinctly convex dorsum results in a “hunched” profile that is characteristic for this species. This profile is similar to that of some species placed in the erstwhile subgenus *Macromischa* Roger (1863).

**Figure 17.**
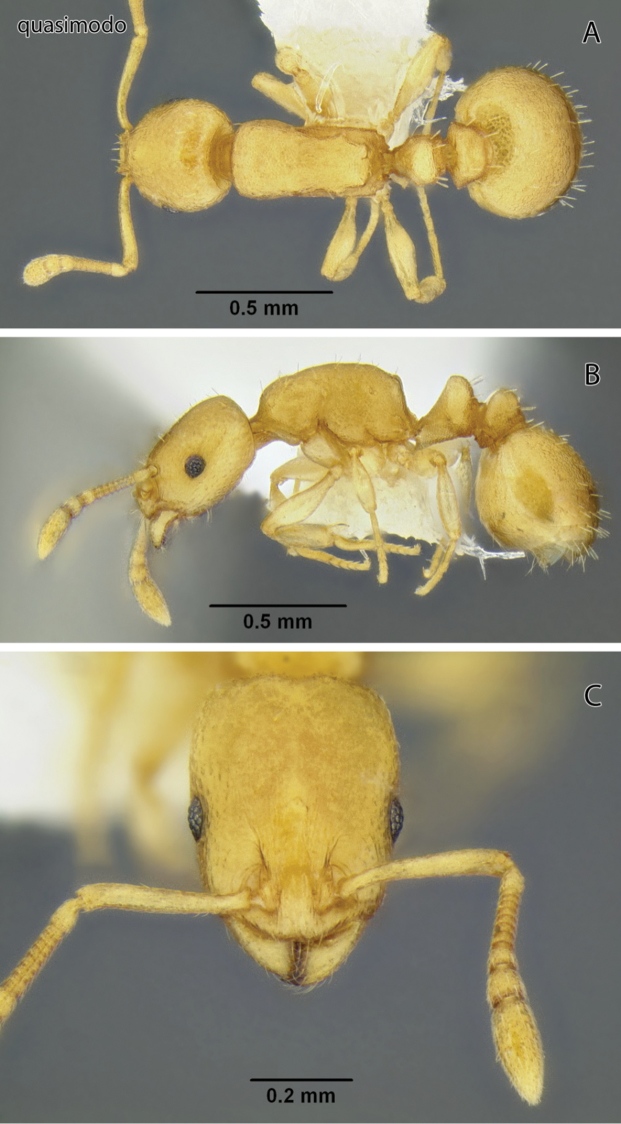
*Temnothorax quasimodo* sp. n., worker (CASENT0005694) **A** Body in dorsal view **B** Body in lateral view **C** Head in full-face view.

Superficially this species resembles *Temnothorax andrei*, a species that is common in similar habitat and was collected in the same litter sample as *Temnothorax quasimodo* (P. S. Ward, pers. comm.). The distinctive mesosomal profile of *Temnothorax quasimodo* will readily distinguish between the two (Figures 17, 21A). The unusual form of both the mesosoma and broadening of postpetiole may indicate that this is a socially parasitic or inquilinous species (Wilson 1984), perhaps in nests of such other species as *Temnothorax andrei*.

We did not observe similar morphological modifications among multiple nest series and hundreds of specimens of *Temnothorax andrei* examined during this study. *Temnothorax quasimodo* was found in a locality where intensive sampling has been carried out, mostly by Philip S. Ward (pers. comm.) and UC Davis entomology students, but the species has been collected only once. We conclude that it represents an extremely infrequently encountered species such as *Lasius atopus* (Cole, 1958a) or *Stigmatomma trigonignathum* (Brown, 1949), although until more material is collected we cannot be certain that the holotype is not a result of a rare environmentally induced malformation.

### Temnothorax rudis

(Wheeler, 1917)

http://species-id.net/wiki/Temnothorax_rudis

Figures 20 – 22

Leptothorax nevadensis subsp. *rudis*Wheeler 1917: 508. Six syntype workers, Camp Curry, Yosemite, California [MCZC] [Images of MCZ_8676 syntype worker examined]. Revived from synonymy and raised to species by Ward 2005: 19.
Leptothorax rudis Wheeler; Stuart and Page 1991: 375. Genetic study (*rudis* implicitly raised to species).
Leptothorax (Myrafant) nevadensis Mackay 2000: 376. *Leptothorax nevadensis rudis* (Wheeler) as junior synonym of *Temnothorax nevadensis*.
Temnothorax rudis (Wheeler); Ward 2005: 19. First combination in *Temnothorax*, revived from synonymy and raised to species.

#### Material examined.

150 series from California, 3 series from Oregon [MLBC, MMPC, PSWC, UCDC].

#### Remarks.

See Ward (2005) for a discussion of this species.

### Temnothorax rugatulus

(Emery, 1895)

http://species-id.net/wiki/Temnothorax_rugatulus

Figures 20 , 22

Leptothorax rugatulus Emery 1895: 321. Syntype workers, South Dakota, Colorado [MSNG] [Images of CASENT0904762 syntype worker examined].
Leptothorax curvispinosus subsp. *rugatulus*Wheeler 1903: 241. *Leptothorax rugatulus* (Emery) as junior synonym of *Leptothorax curvispinosus*.
Leptothorax curvispinosus rugatulus var. *cockerelli*Wheeler 1903: 241. 10 syntype workers, Las Vegas Hot Springs, New Mexico [MCZC] [Images of MCZ_21044 syntype worker examined]. Synonymy by Creighton 1950: 269.
Leptothorax curvispinosus rugatulus var. *annectens*Wheeler 1903: 242. 4 syntype workers, Boulder, Colorado [MCZC] [Images of MCZ_22776 syntype worker examined]. Synonymy by Creighton 1950: 269.
Leptothorax rugatulus (Emery) Wheeler 1917: 509. Revived status as species.
Leptotothorax rugatulus var. *mediorufus*Wheeler 1917: 510. Six syntype workers, Lake Tahoe, California [MCZC] [Images of MCZ_8675 syntype worker examined]. Synonymy by Creighton 1950: 269.
Leptothorax rugatulus subsp. *brunnescens*Wheeler 1917: 510. Syntype workers, Creede, Colorado [MCZC] [Images of MCZ_23661 syntype worker examined]. Synonymy by Mackay 2000: 394.
Leptothorax rugatulus subsp. *cockerelli* Wheeler; Wheeler 1917: 510. *Leptothorax curvispinosus rugatulus* var. *cockerelli* raised to subspecies.
Leptothorax rugatulus subsp. *annectens* Wheeler; Wheeler 1917: 510. *Leptothorax curvispinosus rugatulus* var. *annectens* raised to subspecies.
Leptothorax (Myrafant) rugatulus Emery; M. R. Smith 1950: 30. First combination in *Leptothorax (Myrafant)*.
Temnothorax rugatulus (Emery); Bolton 2003: 272. First combination in *Temnothorax*.

#### Material examined.

65 series from California, 14 series from Arizona, 4 series from Oregon, 4 series from Nevada [MLBC, MMPC, PSWC, UCDC].

#### Remarks.

See Mackay (2000) for a full description of this species.

### Temnothorax wardi sp. n.

http://zoobank.org/C4E708AD-3868-422F-A03C-634E20CA658B

http://species-id.net/wiki/Temnothorax_wardi

**Figure 18.**
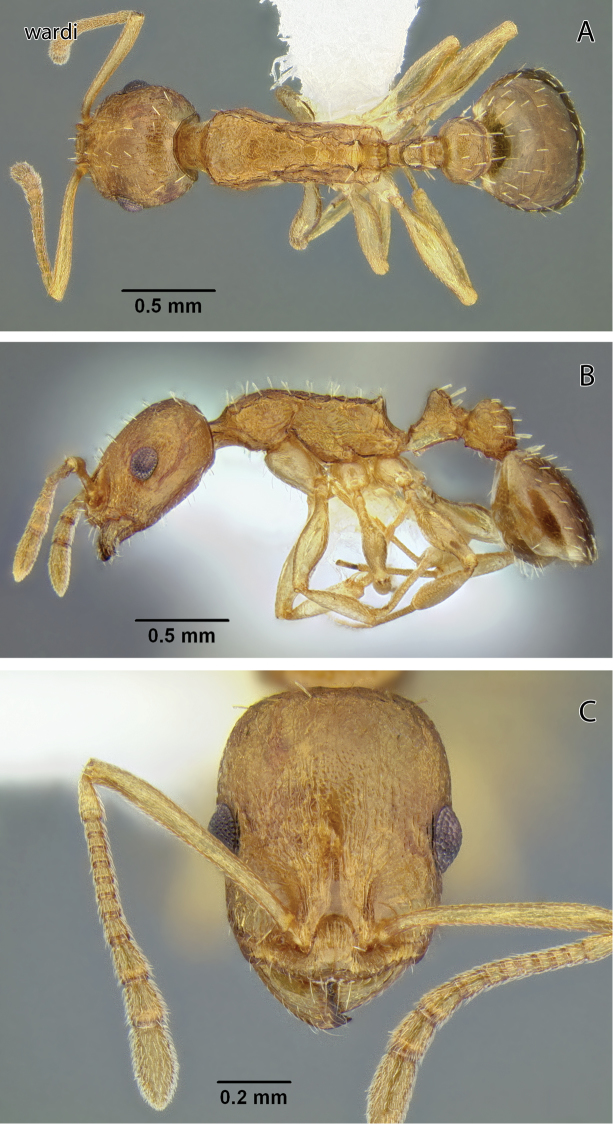
*Temnothorax wardi* sp. n., worker (CASENT0339315) **A** Body in dorsal view **B** Body in lateral view **C** Head in full-face view.

**Figure 19.**
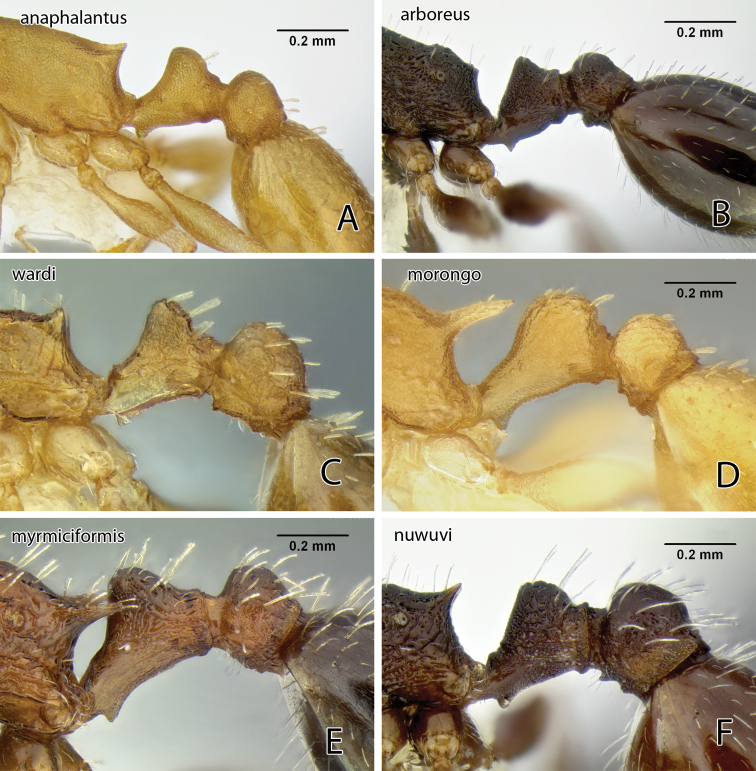
Lateral views focusing on petiole and postpetiole **A** *Temnothorax anaphalantus* sp. n. (CASENT0339237) **B** *Temnothorax arboreus* sp. n. (CASENT0339243) **C** *Temnothorax wardi* sp. n. (CASENT0339315) **D** *Temnothorax morongo* sp. n. (CASENT0103108) **E** *Temnothorax myrmiciformis* sp. n. (CASENT0339323) **F** *Temnothorax nuwuvi* sp. n. (LACMENT299376).

Figures 18 –19 , 24 – 26

Leptothorax sp. BCA-2; Johnson and Ward 2002: 1023.
Temnothorax sp. CA-02; Ward 2005: 68.

#### Diagnosis of worker.

Large, gracile species with large eyes; scape extending slightly beyond posterior margin; side of head, behind eyes, without standing setae; propodeal spines short and triangular; postpetiole extremely wide in dorsal view.

#### Description.

Worker measurements (mm) (3 measured): EL 0.191–0.217 (0.201); HFL 0.725–0.791 (0.761); HFW 0.156–0.182 (0.170); HL 0.792–0.857 (0.827); HW 0.649–0.747 (0.686); IOD 0.538–0.630 (0.578); OMD 0.193–0.224 (0.206); PPW 0.318–0.415 (0.371); PSL 0.068–0.089 (0.079); PTW 0.178–0.210 (0.201); PW 0.441–0.513 (0.472); SL 0.708–0.786 (0.743); WL 1.002–1.124 (1.059). Indices: CI 80.7–87.2 (82.9); FI 106–114 (111); OI 23.9–25.3 (24.3); PI 179–198 (184); PSI 8.59–10.5 (9.57); SI 89.1–91.7 (89.8).

Head longer than broad, margins subparallel, broadly rounded into transverse posterior margin. Antenna 12-segmented and scape extending slightly beyond posterior margin of head; three-segmented apical club poorly defined. Eyes moderately convex and without short setae arising from between ommatidia; IOD 2.77–2.90 × EL; EL 0.96–1.01 × OMD. Medial carina of clypeus sharply defined, as are 2–3 short carinae on either side. Malar area with several well–spaced longitudinal rugae that curve mesad of eye to slightly behind level of upper eye margin; similar rugae curve mesad above antennal fossae. Frons and vertex moderately shiny and reticulate and with scattered poorly defined longitudinal rugae. Dorsum with numerous short, fully erect, slightly flattened setae, but none along margins of head in frontal view; venter with sparse, finer setae about as long as those of frons.

Mesosoma slender, WL 2.19–2.29 × PW; dorsal profile nearly flat from anterior edge of mesonotum to base of propodeal spines. Propodeal spines short and stout, less than half as long as distance between their bases. Pronotal dorsum and side coarsely corrugated/rugoreticulate; remainder of dorsum with variable degrees of corrugation and/or irregular longitudinal rugae; interspaces moderately shiny and reticulate; mesepisternum and propodeum side with well–spaced, irregular coarse rugae and densely reticulate interspaces. Hind femur 4.31–4.65 times longer than wide in dorsal view. Dorsum with 15–20 fully erect flattened, blunt–tipped setae that are longer than those of frons.

Petiole node high and subacute in profile; subpetiolar process short and blunt; postpetiole node robust, high and broadly rounded in profile, about twice as wide as petiole node; Posterior face of petiole node coarsely areolate, postpetiole node contiguously punctate only.

Gaster in dorsal view 1.83–2.41 times as wide as postpetiole node; first tergum smooth and shiny, with sparse piligerous punctures, the setae suberect, slightly flattened, and blunt-tipped.

Head and body light reddish brown, gaster darker.

Gyne and male unknown.

#### Material examined.

Holotype worker, U.S.A.: CALIFORNIA: San Bernardino Co.: San Savaine Road, Lytle Creek, 1395 m, 34.1993°, -117.4815°, 3.viii.2004 (J. des Lauriers), ex pitfall trap, burned scrub oak chaparral (CASENT0339315) [UCDC].

Paratypes. Same data as holotype, 1 worker (CASENT0339315) [UCDC].

Non-paratypic material examined. U.S.A.: CALIFORNIA: Los Angeles Co.: Mount Baldy Road, Hogback, 1165 m, 34.220°, -117.668°, 25.i.2006 (J. des Lauriers) *Ceanothus* chaparral, 1 worker (CASENT0339317) [LACM]; San Diego Co.: Elliot Reserve 1 km E Miramar, 180 m, 32.88°, -117.1°, 2.ix.2000 (P. S. Ward, #PSW14263-5) ex nest middens of *Forelius* sp., chaparral, 2 workers (CASENT0339318) [UCDC]; San Diego Co.: 3 km S Santa Ysabel, 33.08°, -116.67°, 10.v.1987 (M. Trepanier) ground forager, chaparral/*Quercus virginiana* (sic) woodland, 1 worker (CASENT0005691) [UCDC]; MEXICO: BAJA CALIFORNIA: 28 km E Ensenada, 750 m, 31.8833°, -116.3000°, 27.iv.1998 (P. S. Ward, #PSW13576-8) ex nest middens of *Forelius* sp., chaparral, 1 worker (CASENT0339319) [UCDC].

#### Etymology.

This species is dedicated to Phil Ward, who provided so much of the material utilized in this paper.

#### Remarks.

The combination of flat mesosomal dorsum, short and upward-directed propodeal spines with extremely swollen postpetiole will separate this species from all other western *Temnothorax*. In dorsal view the relatively closely spaced propodeal spines and the postpetiole, which is almost twice as wide as the petiole, are especially conspicuous. (PI 179–198; Figure 18A).

The known specimens have been collected in pitfall traps in chaparral and from nest middens of *Forelius* ants.

### Temnothorax whitfordi

(Mackay, 2000)

http://species-id.net/wiki/Temnothorax_whitfordi

Figure 22

Leptothorax (Myrafant) whitfordi Mackay 2000: 421. Holotype worker, 5.3 km SE Sitting Bull Falls, Guadalupe Mountains, Eddy Co., New Mexico [holotype worker in MCZC and multiple paratype worker specimens in various institutions] [Paratype in UCDC examined].
Temnothorax whitfordi (Emery); Bolton 2003: 272. First combination in *Temnothorax*.

#### Material examined.

3 series from California, 5 series from Arizona [PSWC, UCDC].

#### Remarks.

See Mackay (2000) for a full description of this species.

### Key to California species of *Temnothorax*

Below we provide a worker-based key to the 20 species of *Temnothorax* known from California, plus two additional species (*Temnothorax nuwuvi*, *Temnothorax carinatus*) known from neighboring states to the east.

In California, *Temnothorax* can be most easily confused with *Leptothorax*, *Tetramorium* and *Myrmica*, but all these genera have at least six mandibular teeth. Additionally, *Tetramorium* and *Myrmica* possess mid- and hind tibial spurs. In western North America, workers of *Temnothorax* can be recognized by a combination of a waist consisting of two segments (petiole and postpetiole); postpetiole attached to anterior face of abdominal segment 4 (first gastral segment); absence of promesonotal suture from mesosoma; antennae 11- or 12-segmented and with differentiated 3-segmented club; eyes present and consisting of at least several facets; frontal carinae not laterally expanded or extending posteriorly past the eye; presence of median clypeal carina; clypeus elevated slightly above mandibles in profile view, not an anteriorly projecting lobe that fits tightly over the base of the mandibles; mandibles with five teeth; propodeal spines present at least as distinct tubercles and tibial spurs on middle and hind legs absent. A user-friendly key to ant genera of North America is available in Fisher and Cover (2007).

**Table:** 

1	Antenna 11-segmented (Figure 6C)	2
–	Antenna 12-segmented (Figure 5C)	5
2(1)	Propodeal spines longer than 0.5 × distance between their bases, PSI 19–30; pronotal dorsum coarsely areolate-rugose or at least irregularly rugose (Figures 6A, 20E, 20F, 22I)	3
–	Propodeal spines dentiform, always much less than 0.5 × distance between their bases, PSI 6–10; pronotal dorsum dull and reticulate, without coarse areolae or rugae (Figures 22G, 22J)	4
3(2)	Petiole node profile subacute dorsally (Figure 20F), with dorsal face rounding evenly into posterior face of node; petiole 0.97–1.04 times longer than high in profile when length is measured on a plane parallel to the venter of the petiole, from the anterior flange of the peduncle to the posterior margin of the petiole and the height is measured from the top of the node to the ventral margin of the petiole in a plane orthogonal to the length measurement; metafemur slender, 4.0–4.7 times longer than wide in dorsal view; ground-nesting species, occasionally aboreal	*Temnothorax rugatulus* (Wheeler)
–	Petiole node broadly rectangular (Figure 20E), with an oblique angle between the dorsal and posterior faces of node; petiole 1.04–1.18 times longer than high in profile; metafemur stout, 3.5–4.2 times longer than wide in dorsal view; exclusively arboreal nesting species	*Temnothorax caguatan* sp. n.
4(2)	Head, especially posteriorly, with extensive smooth shiny areas; promesonotum often with smooth or weakly sculptured areas (Figure 22J); summit of petiole node blunt in profile	*Temnothorax whitfordi* (Mackay)
–	Dorsa of head and mesosoma reticulate and dull (Figure 22G); summit of petiole node acute in profile	*Temnothorax oxynodis* (Mackay)
5(1)	Eye round to oval (Figure 21G)	6
–	Eye elongate, approximately kidney-shaped (Figure 21H)	*Temnothorax obliquicanthus* (Cole)
6(5)	Mesosomal dorsum with metanotal groove impressed, pronotum distinctly higher than the base of propodeum (Figures 10B, 13B)	7
–	Mesosomal dorsum without a distinctly impressed metanotal groove (Figures 1B, 9B)	8
7(6)	Dorsum of head and mesosoma slightly shiny and finely reticulate, without longitudinal rugae (Figures 13A, 13C)	*Temnothorax paiute* sp. n.
–	Dorsum of head and mesosoma shiny between fine to coarse longitudinal rugae (Figures 10A, 10C)	*Temnothorax myrmiciformis* sp. n.
8(6)	First gastral tergum moderately to slightly shiny, most of disc distinctly finely sculptured (Figure 23B)	9
–	First gastral tergum smooth and shiny, appearing polished, devoid of sculpture other than scattered minute piligerous punctures (Figure 23A)	10
9(8)	Head and mesosoma reticulate and dull, without longitudinal rugae; propodeal spines broad at base (in profile) and slightly less than the distance between their bases, PSI 14–17 (Figures 2A–C)	*Temnothorax anaphalantus* sp. n.
–	Head and mesosoma reticulate and dull between distinct longitudinal rugae; propodeal spines slender and distinctly longer than distance between their bases, PSI 22–29 (Figures 9A–C)	*morongo* sp. n.
10(6)	Dorsal mesosomal profile flat or weakly convex; mesosoma slender and distance from dorsal mesonotal margin to base of mesocoxa 0.30–0.34 × WL; eye oval and EL at least 0.8 × OMD (Figures 21A, 21E, 21G)	11
–	Dorsal mesosomal profile distinctly convex; mesosoma compact and distance from dorsal mesonotal margin to base of mesocoxa 0.5 × WL; eye round and EL about 0.7 × OMD (Figures 17A–C)	*Temnothorax quasimodo* sp. n.
11(10)	Petiole node triangular to subtriangular in profile (Figures 19B, 21B, 21C and 21E)	12
–	Petiole node rounded to rectangular in profile (Figures 19F, 20B, 20D, 21A, 21D and 21F)	15
12(11)	Propodeal spines as long as, or longer than, distance between their bases, PSI 20–26 (Figures 21C, 22C); arboreal species nesting in galls and dead twigs	*Temnothorax gallae* (M.R. Smith)
–	Propodeal spines distinctly shorter than the distance between their bases, PSI 10–16 (Figures 19B, 21B, 21E, 22B, 22F); arboreal or ground nesting species	13
13(12)	Color dark brown; propodeal spines reduced to slight angles, much shorter than distance between their bases, PSI 4–5 (Figures 5A, 19B); arboreal nesting species	*Temnothorax arboreus* sp. n.
–	Color yellowish brown to brown; propodeal spines present and acute in profile, up to one-half the distance between their bases, PSI 10–16 (Figures 21B, 21E, 22B, 22F)	14
14(13)	Head reticulate and with variably extensive smooth shiny areas (Figure 23D); mesosomal dorsum weakly to moderately reticulate, pronotum often shinier than propodeal dorsum (Figure 22F); ground nesting species	*Temnothorax nitens* (Emery)
–	Head uniformly reticulate but with smooth shiny median stripe (Figure 23C); entire mesosomal dorsum strongly reticulate (Figure 22B)	*Temnothorax chandleri* (Mackay)
15(11)	Propodeal spines as long as, or slightly shorter than the distance between their bases, PSI 15–24; light to dark brown ground nesting species	16
–	Propodeal spines distinctly shorter than the distance between their bases, PSI 4–17; either arboreal or ground nesting; color variable	17
16(15)	Mesosomal dorsum reticulate, often appearing lineoreticulate, sometimes with weak longitudinal rugae (Figure 22E); petiole node, in profile, less robust (Figure 21D)	*Temnothorax nevadensis* (Wheeler)
	Note: As presently understood, *Temnothorax nevadensis* is a variable species, occurring over a wide range of habitats (Wheeler and Wheeler 1986), and its taxonomic status is in need of further study (see also Ward 2005: 16).	
–	Mesosomal dorsum reticulate between moderately coarse longitudinal rugae and usually promesonotum partly coarsely areolate (Figure 22H); petiole node, in profile, more robust (Figure 20D)	*Temnothorax rudis* (Wheeler)
17(15)	Postpetiole wide, PPW 1.47–1.98 × PTW (Figures 11A, 18A, 22A, 22D)	18
–	Postpetiole narrow, PPW 1.23–1.43 × PTW (Figure 14A)	21
18(17)	Postpetiole narrow relative to petiole, PPW 1.47–1.76 × PTW; mesosomal dorsum reticulate and variously rugulose in part, but not coarsely areolate with microreticulate interspaces (Figures 22A, 22D)	19
–	Postpetiole wide relative to petiole, PPW 1.79–1.98 × PTW; pronotal disc and dorsal face of propodeum coarsely areolate with microreticulate interspaces (Figure 18A)	*Temnothorax wardi* sp. n.
19(18)	Postpetiole dorsum reticulate and dull (Figures 22A, 22D); head shiny and with weakly defined fine rugulae, but extensive areas without rugae	20
–	Postpetiole shiny between weak punctures and with medial impunctate area (Figure 11A); head with distinct coarse longitudinal rugae, interspaces variably reticulate (Figure 11C)	*Temnothorax nuwuvi* sp. n.
20(19)	Mesosomal dorsum with variably extensive smooth areas, otherwise reticulate (Figure 22D); propodeal spines acute in profile and, when viewed obliquely from above, surface between them nearly flat; dark brown species	*Temnothorax neomexicanus* (Wheeler)
–	Mesosomal dorsum reticulate (Figure 22A); propodeal spines robust in profile, nearly right angular at tips and, when view obliquely from above, surface between them approximately semicircular; light brownish yellow species	*Temnothorax carinatus* (Cole)
21(17)	Cephalic venter with scattered fine setae; frons, in profile, with short setae present along entire length, from base of clypeus to posterior margin (Figure 21G)	*Temnothorax andrei* (Emery)
–	Cephalic venter without setae; frons, in profile, with few or no setae, but with 2 on lower vertex and 4 across posterior margin (Figure 14B)	*Temnothorax pseudandrei* sp. n.

**Figure 20.**
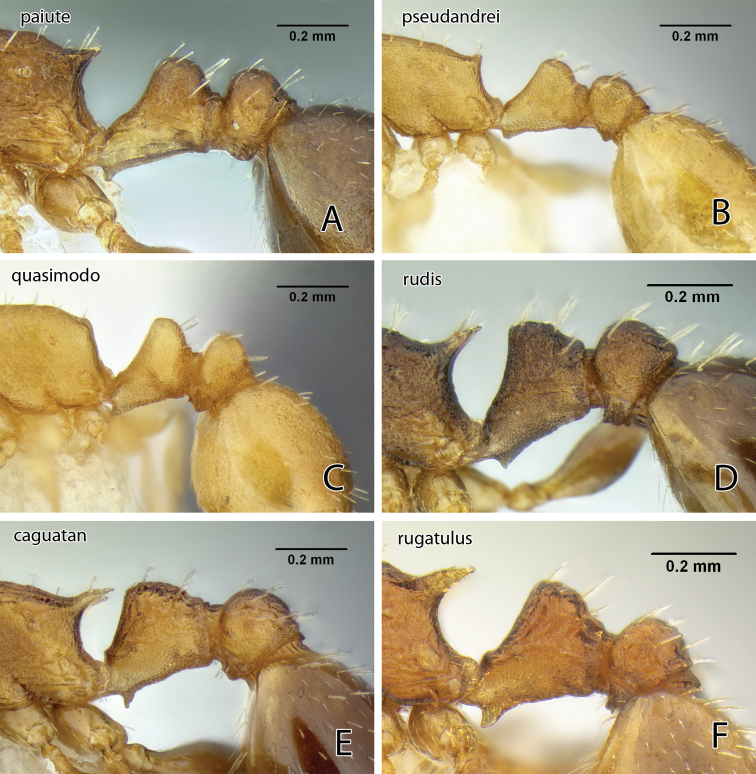
Lateral views focusing on petiole and postpetiole **A** *Temnothorax paiute* sp. n. (CASENT0005932) **B** *Temnothorax pseudandrei* sp. n. (CASENT0005693) **C** *Temnothorax quasimodo* sp. n. (CASENT0005694) **D** *Temnothorax rudis* **E** *Temnothorax caguatan* sp. n. (CASENT0339286) **F** *Temnothorax rugatulus*.

**Figure 21.**
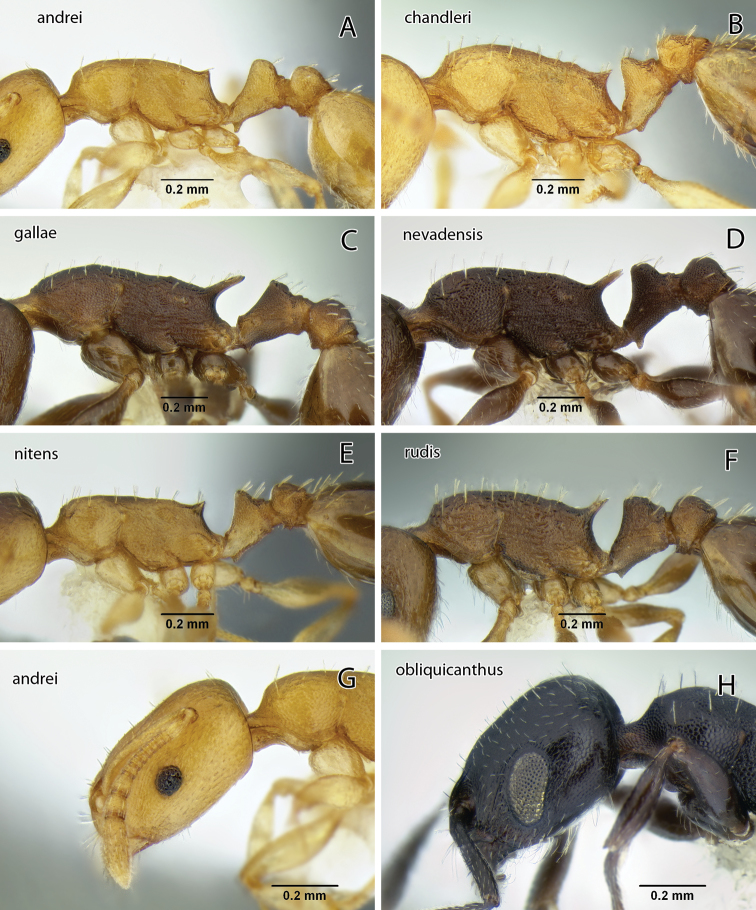
**A–F** Lateral views focusing on mesosoma **A** *Temnothorax andrei* **B** *Temnothorax chandleri* **C** *Temnothorax gallae* **D** *Temnothorax nevadensis* **E** *Temnothorax nitens* **F** *Temnothorax rudis* **G–H** Lateral views focusing on head **G** *Temnothorax andrei* **H** *Temnothorax obliquicanthus*.

**Figure 22.**
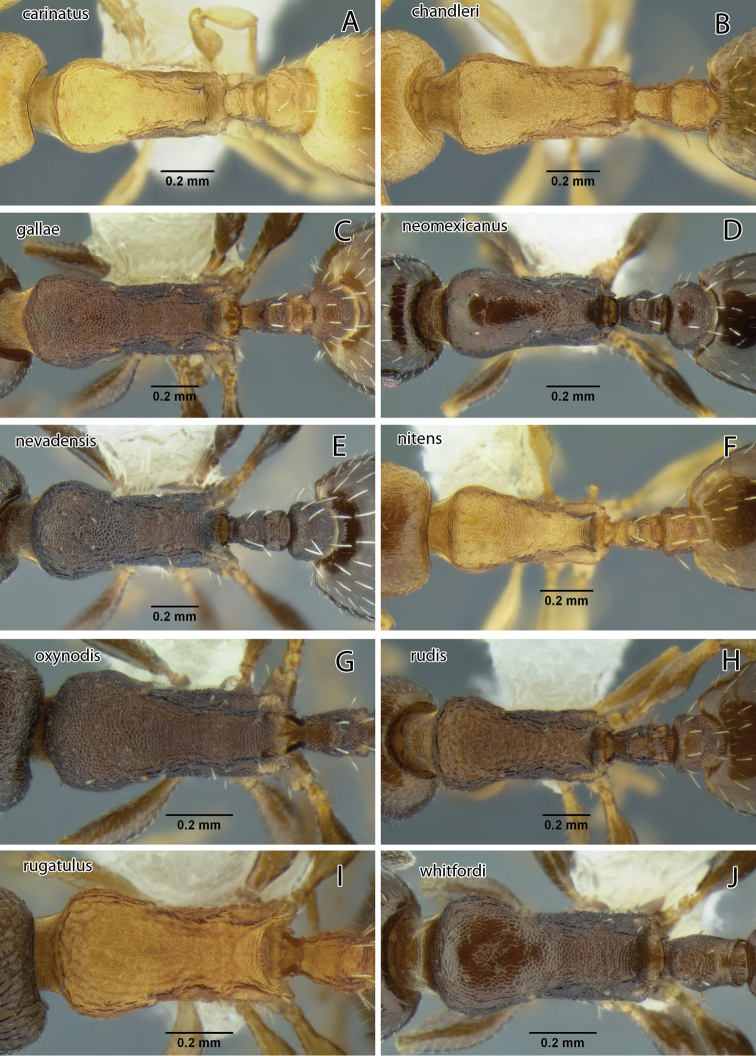
Dorsal views focusing on mesosoma **A** *Temnothorax carinatus* **B** *Temnothorax chandleri* **C** *Temnothorax gallae* **D** *Temnothorax neomexicanus* **E** *Temnothorax nevadensis* **F** *Temnothorax nitens* **G** *Temnothorax oxynodis* **H** *Temnothorax rudis* **I** *Temnothorax rugatulus* **J** *Temnothorax whitfordi*.

**Figure 23.**
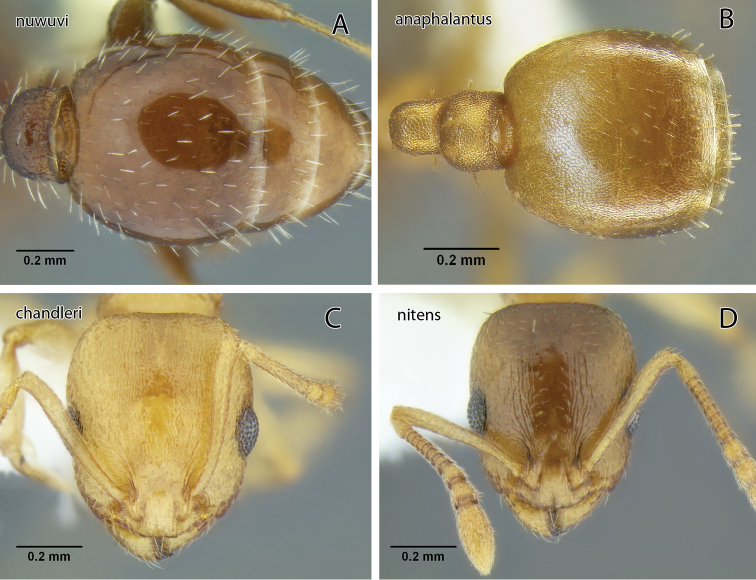
**A–B** Dorsal views focusing on gaster **A** *Temnothorax nuwuvi* sp. n. (CASENT0172608) **B** *Temnothorax anaphalantus* sp. n. (CASENT0339238) **C–D** Full-face views of the head **C** *Temnothorax chandleri* **D** *Temnothorax nitens*.

**Figure 24. F24:**
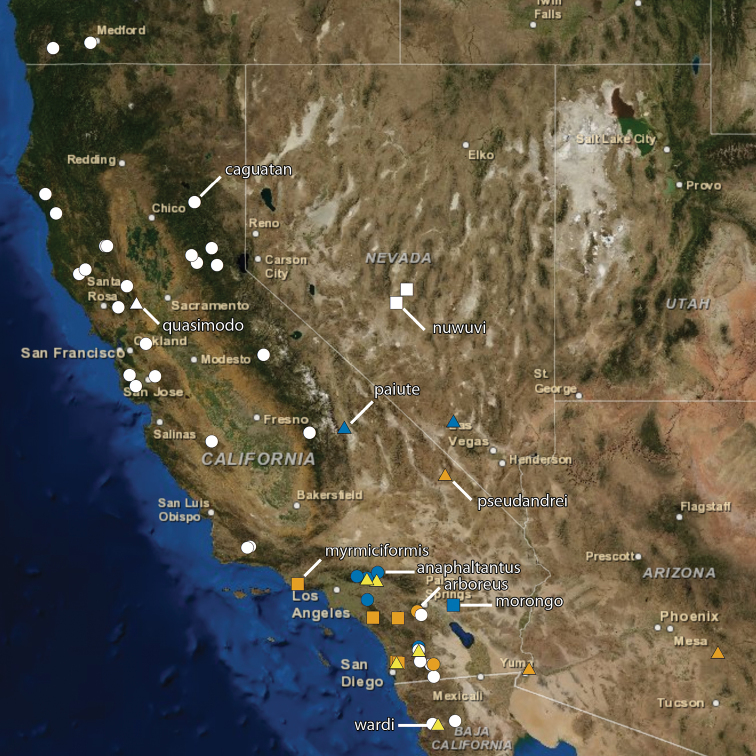
Northern part of recorded distribution of the new species, focusing on southern Oregon, California and Nevada. White circles represent records of *Temnothorax caguatan*; blue circles – *Temnothorax anaphalantus*; orange circles – *Temnothorax arboreus*; white rectangles – *Temnothorax nuwuvi*; blue rectangles – *Temnothorax morongo*; orange rectangles – *Temnothorax myrmiciformis*; white triangle – *Temnothorax quasimodo*; blue triangles – *Temnothorax paiute*; orange triangles – *Temnothorax pseudandrei*; yellow triangles – *Temnothorax wardi*.

**Figure 25. F25:**
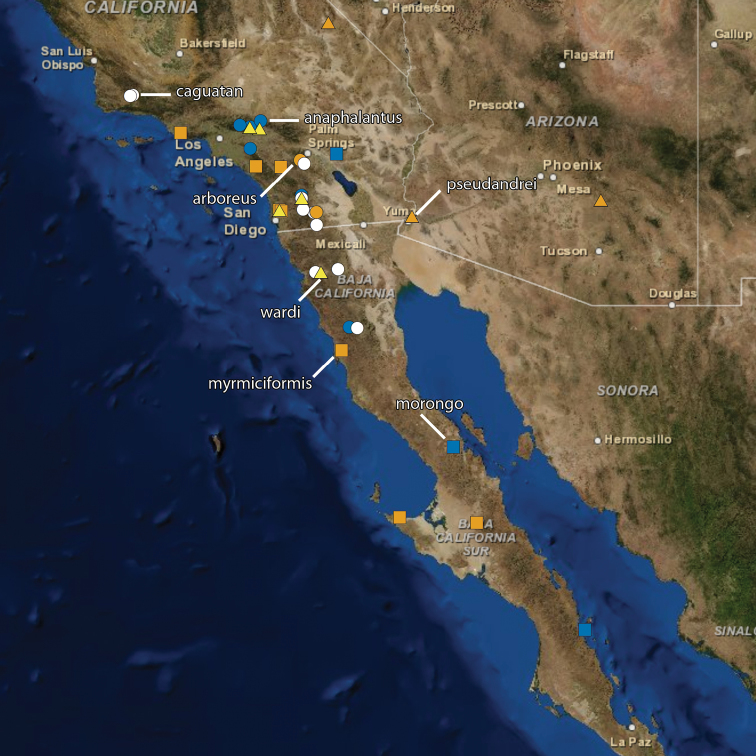
Southern part of recorded distribution of the new species, focusing on southern California, Baja California and Arizona. White circles represent records of *Temnothorax caguatan*; blue circles – *Temnothorax anaphalantus*; orange circles – *Temnothorax arboreus*; blue rectangles – *Temnothorax morongo*; orange rectangles – *Temnothorax myrmiciformis*; orange triangles – *Temnothorax pseudandrei*; yellow triangles – *Temnothorax wardi*.

**Figure 26.**
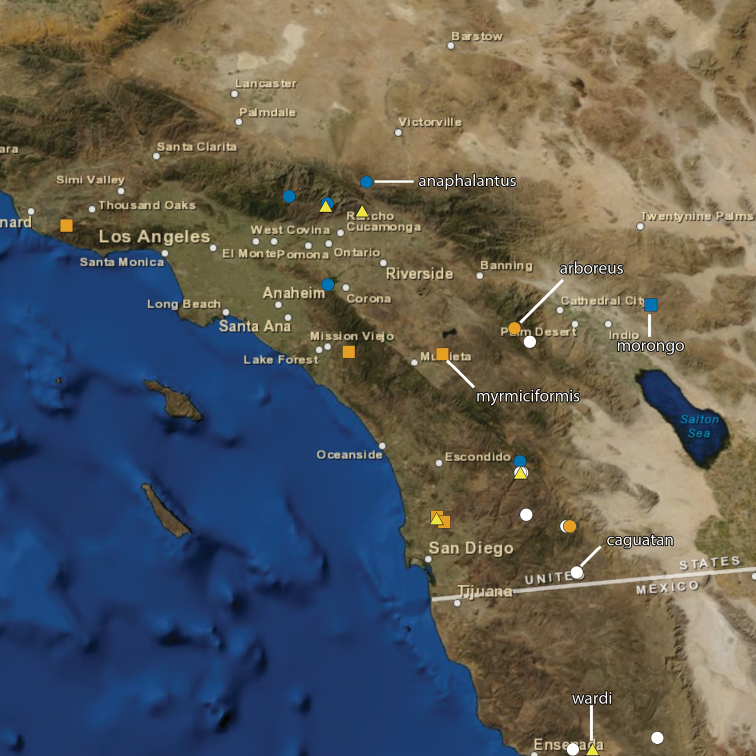
Distribution of the new species, focusing on southern California where many species co-occur. White circles represent records of *Temnothorax caguatan*; blue circles – *Temnothorax anaphalantus*; orange circles – *Temnothorax arboreus*; blue rectangles – *Temnothorax morongo*; orange rectangles – *Temnothorax myrmiciformis*; yellow triangles – *Temnothorax wardi*.

## Supplementary Material

XML Treatment for Temnothorax anaphalantus

XML Treatment for Temnothorax andrei

XML Treatment for Temnothorax arboreus

XML Treatment for Temnothorax caguatan

XML Treatment for Temnothorax carinatus

XML Treatment for Temnothorax chandleri

XML Treatment for Temnothorax gallae

XML Treatment for Temnothorax morongo

XML Treatment for Temnothorax myrmiciformis

XML Treatment for Temnothorax neomexicanus

XML Treatment for Temnothorax nevadensis

XML Treatment for Temnothorax nitens

XML Treatment for Temnothorax nuwuvi

XML Treatment for Temnothorax oxynodis

XML Treatment for Temnothorax paiute

XML Treatment for Temnothorax pseudandrei

XML Treatment for Temnothorax quasimodo

XML Treatment for Temnothorax rudis

XML Treatment for Temnothorax rugatulus

XML Treatment for Temnothorax wardi

XML Treatment for Temnothorax whitfordi

## Appendix

Specimen data associated with the new species. (doi: 10.3897/zookeys.372.6039.app) File format: Text file (txt).

**Explanation note:** This text file contains data for all specimens belonging to the new species described in this article.
